# Towards a replacement therapy for stimulant betel quid dependence: A proof of concept study

**DOI:** 10.1111/adb.13371

**Published:** 2024-02-06

**Authors:** Peter G. Osborne, Ragavendra Rao Pasupuleti, Chien‐Hung Lee, Vinoth Kumar Ponnusamy

**Affiliations:** ^1^ Baongong Agriscience Center Fengtian Taiwan; ^2^ Department of Medicinal and Applied Chemistry Kaohsiung Medical University (KMU) Kaohsiung City Taiwan; ^3^ Research Center for Precision Environmental Medicine Kaohsiung Medical University (KMU) Kaohsiung City Taiwan; ^4^ Department of Public Health, College of Health Sciences Kaohsiung Medical University (KMU) Kaohsiung City Taiwan; ^5^ Department of Medical Research Kaohsiung Medical University Hospital (KMUH) Kaohsiung City Taiwan; ^6^ Department of Chemistry National Sun Yat‐sen University (NSYSU) Kaohsiung City Taiwan; ^7^ Program of Aquatic Science and Technology, College of Hydrosphere Science National Kaohsiung University of Science and Technology (NKUST) Kaohsiung City Taiwan; ^8^ Present address: Department of Chemistry Indiana University Bloomington IN USA

**Keywords:** addiction, *Areca Catechu*, dependence, Mg(OH)_2_, Oral cancer, UHPLC–MS/MS

## Abstract

Stimulant betel quid (SBQ) containing *Piper betle* leaf (L), green unripe *Areca catechu* nut (AN) and the alkalizing agent, slaked lime, is an addictive, carcinogenic stimulant, with no pharmacotherapy, chewed by millions of people in the Asia/Pacific region. We compared the in vivo physiological profile of chewing (1) non‐stimulant *P. betle* leaf+AN (LAN), (2) SBQ utilizing slaked lime and (3) a novel SBQ utilizing Mg(OH)_2_, as an alkalizing agent, by measuring physiological parameters of intoxication and these were correlated with in vitro levels of alkaloids measured by UHPLC–MS/MS. Chewing LAN, which contains high levels of arecoline, had no stimulatory physiological effect. Chewing SBQ containing slaked lime or novel SBQ containing Mg(OH)_2_, induced equivalent stimulatory physiological responses. In vitro, slaked lime hydrolyzed muscarinic esters in LAN while Mg(OH)_2_ did not. The physiological stimulation induced by chewing both SBQ and the lack of physiology to chewing LAN can be explained by changes in lipid solubility of phytochemicals induced by mouth pH during chewing of basic SBQ or acidic LAN. Since antiquity people have added slaked lime to SBQ to enhance absorption of phyto‐chemicals across oral membranes to stimulate physiology. The same physiological changes can be induced by substituting slaked lime for less physically and chemically destructive bases. If attitudes regarding SBQ dependence can advance towards the more progressive attitudes already used to help smokers quit tobacco, modern chemistry has the potential to make chewing SBQ safer and quitting programs may become more accessible and efficacious.

## Abstract 2

Chewing *Piper betle* leaf and green unripe *Areca catechu* nut (LAN), which contains high levels of arecoline, had no stimulatory physiological effect. Chewing stimulant betel quid (SBQ) containing LAN+slaked lime or SBQ containing LAN+Mg(OH)_2_, induced equivalent stimulatory physiological responses. Since antiquity people have added slaked lime to LAN to enhance absorption of phyto‐chemicals across oral membranes to stimulate physiology. The same physiological changes can be induced by substituting slaked lime for less physically and chemically destructive bases.

## 1 INTRODUCTION

Areca nut (AN) is the fruit of the *Areca catechu* palm and can be chewed by itself or may be combined with other plants and chemicals to be chewed as a quid. The International Agency for Research on Cancer 2004 monograph (IARC 2004 monograph)^1^ formally recognized that chewing betel quid (BQ) containing slaked lime, AN and frequently *Piper betle* leaf or inflorescence is carcinogenic to humans. In hindsight it is obvious that in the sections of this monograph devoted to human physiology and pharmacology, the terms AN and BQ were erroneously often used interchangeably (see supporting information, P235 section 5.4, P236) and in so doing this publication appears to be the origin of a long standing historical scientific confusion of the physiology attributed to these two distinctly different types of chewable quids. In addition, the IARC 2004 monograph evaluated AN as carcinogenic to humans on the basis of five positive and numerous negative rat, mice and hamster toxicology studies and several epidemiological studies reporting the presence of precancerous lesions.^1^ However, because of the ambiguity arising from the interchangeable use of AN and BQ in the IARC 2004 monograph it is extremely difficult to be confident that the epidemiology of AN consumption was accurately categorized in the monographs assessment (see supporting information).

The uncritical, universal acceptance of the scientific evaluation presented in IARC 2004 monograph, and subsequent demonstrations that extracts of AN and arecoline, the most abundant muscarinic ester in unripe AN^2^, ^3^, ^4^, ^5^ are cytopathological in a number of experimental models^1^, ^6^, ^7^ influenced, and still influences, medical and research attitudes and public health strategies and initiatives, and the small number of government, community and hospital counselling programs available, to be designed around educating chewers to abstain from chewing AN, BQ and BQ + Tobacco (BQ + T). It would not be contentious to say that this policy of abstinence has not been overly successful. Young people and students still chew,^8^, ^9^ pregnant mothers still chew,^10^ there are no reports of obvious decreases in the incidence of chewing and the high incidence of oral cancer associated with chewing BQ is a serious health problem for many Asian and Pacific countries.^11^, ^12^ It has been 20 years since the 2004 IARC monograph and today there remains no pharmacological replacement therapy for BQ or BQ + T substance use disorders (SUD) and no WHO strategies for minimizing dependence.^13^

Pharmacological replacement therapies to treat drug based SUD are uncommon, being limited to the opiates SUD and tobacco SUD. Once standardized these treatments are significantly more effective than abstinence or counselling alone in reducing consumption, facilitating quitting and decreasing negative health impacts of these SUD.^14^, ^15^, ^16^ However, the development and eventual use of pharmacological replacement therapies requires a philosophical change in attitude away from an imperative of user safety through abstinence, and to accept, that the consumption of similar drugs, in the case of morphine and heroin dependence,^15^ or the same drug, in the case of nicotine dependence,^16^ is not a negative, but in fact a positive, if it results in increased efficacy of completing quitting programs or reducing harm to the user. In regard to BQ, attempts to develop pharmacological replacement therapies face the philosophical problem that the current operational dogma is that exposure to extracts of AN or arecoline from AN is to be avoided at all costs and abstinence or, in very exceptional cases, abstinence combined with counselling, is the recommended treatment.

The IARC 2004 monograph failed to distinguish between AN and BQ and because it is an enormously influential publication this error has come to dominate the scientific and health narratives about chewing AN containing quids. Currently the scientific literature does not recognize that BQ can be usefully categorized into two functionally, mutually exclusive types: nonstimulant BQ (nonSBQ) and stimulant BQ (SBQ). Paradoxically this useful division is well known to chewers. NonSBQ are preparations containing a wide variety of herbs, spices, and importantly also AN and *P. betle* leaf (L), which, in Sri Lanka, India and Pakistan, are usually chewed after meals as mouth fresheners that, having antibacterial properties, have traditionally been use to promote oral hygiene.^17^ In Sri Lanka, epidemiological studies show that chewing nonSBQ containing AN is not dependence forming.^18^, ^19^ In Taiwan, as in most countries, people do not regularly chew AN alone. As referenced above, in the early scientific literature the terms betel nut, AN and BQ were frequently used interchangeably^19^ and this, coupled with the high levels of muscarinic esters measured in AN extracts,^2^, ^3^, ^4^, ^5^ has produced a general scientific acceptance that chewing AN will induce a pharmacologically based physiological stimulation. However in contrast to what has long been assumed scientifically, a small study on five people in Taiwan showed that the chewing of unripe AN together with *P. betle* leaf (LAN), in the absence of slaked lime, is without measurable mental or physiologically effect on face temperature, heart rate and facial flushing.^5^ In humans, intravenous infusion of 5 mg of arecoline over 30 min, a dose less than that contained in AN when chewing LAN, induced nausea, dizziness, dry mouth, hypothermia and a gradual increase in heart rate.^20^ The difference between the physiological response to intravenous infusion and chewing suggests that the alkaloids in LAN are not functional after absorption or are not absorbed into cells during chewing and this indicates that AN and LAN, by definition, can be considered forms of nonSBQ. This is potentially advantageous for treatment of SBQ SUD and confirming this finding in a larger group of chewers is the first step towards determining the suitability of including LAN as a culturally appropriate placebo to alleviate chewing cravings in SBQ quitting programs.

In contrast to nonSBQ, SBQ also contains AN, invariably *P. betle* leaf but critically must contain slaked lime, which is predominantly Ca(OH)_2,_ NaOH and smaller amounts of KOH, that act as hydrolyzing agents for phyto‐compounds during chewing^4^, ^5^ and facilitates increases in the concentration of unbound, unionized alkaloids and passive penetration across cell membranes of the mouth via pKa and pH interactions.^21^ SBQ containing slaked lime is regularly chewed by more than 5 million people in Southern China, Taiwan, Papua New Guinea and West Papua.^5^ SBQ is dependence forming as determined by every previous iteration of epidemiological instrument for the assessment of dependence,^19^ and most recently by DSM‐V criteria.^22^ Tobacco and spices may also be added to this basic SBQ (SBQ + T) and this combination is regularly chewed by hundreds of millions of people in India, Asia and the Pacific.^19^ Habitual consumption of SBQ or SBQ + T is associated with increased risk of oral and pharyngeal carcinoma and pathology in numerous organs.^23^, ^24^ SBQ + T combines the dependences and health risks of SBQ with those of smokeless tobacco consumption.^25^, ^26^, ^27^

Ample epidemiological research demonstrates that the risk of oral cancer increases with amount of SBQ and SBQ + T chewed and the number of years people chew.^22^, ^28^ Yet very little research effort focuses on developing strategies to decrease SBQ dependence, which if effective, will decrease an individual's exposure to SBQ and reduce the risk of oral cancer. In Taiwan and other countries, counselling based cessation programs for SBQ are rare,^13^ result in improved health education about SBQ chewing, rarely have long term follow up and have small impact on quitting of chewing.^29^, ^30^ The most effective method to treat SBQ SUD requires the development of a culturally sensitive replacement for SBQ with slaked lime. In preliminary tests for this paper we measured the physiological effect of replacing slaked lime in SBQ with bases of lower pH and focused our research on the most promising replacement base, magnesium hydroxide (Mg(OH)_2_).

This paper presents the data from a small scale proof of concept pilot study that compares the physiological and biochemical effects of a SBQ containing a novel hydrolyzing agent, Mg(OH)_2_, a compound approved by the FDA for human consumption^31^ against SBQ containing slaked lime, a compound not approved for human consumption, with the goal of finding a less orally destructive alkaline paste that retains properties to facilitate psychostimulant and physiological activation that could be tested as a theoretical replacement for slaked lime in SBQ. We did not evaluate degrees of CNS stimulation or dependency related processes. In vitro, we simulated the mixing environment of the mouth and measured, by UHPLC–MS/MS, the effects of the major constituent of slaked lime, Ca(OH)_2_, and the novel hydrolyzing agent, Mg(OH)_2_, on the levels of muscarinic esters, carboxylic acid GABA uptake inhibitor metabolites and the mutagenic compound, nitrosoguvacoline, from commercially available LAN and AN extracted into a solution ionically equivalent to human saliva.

In human studies, the physiological effect of three chewing regimes: (1) NonSBQ, containing *P. betle* Leaf and AN (LAN); (2) SBQ containing LAN + slaked lime and (3) novel SBQ containing LAN + Mg(OH)_2_ was quantified by measuring HR, blood pressure, forehead temperature, pupil dilation and facial flushing in social chewers and these physiological responses were correlated with in vitro levels of alkaloids. Healthy social chewers, rather than dependent chewers, were employed so as to avoid confounding effects of adaptation of physiological responses due to chronic exposure to chewing SBQ. In vitro a mechanical chewer was used to masticate the three chewing regimes with human saliva and the pH of final quid solution was measured.

## 2 MATERIALS AND METHODS

A description of reagents, materials and standard solutions are presented in supporting information.

### 2.1 UHPLC–MS/MS instrumental conditions

The concentrations of arecoline, guvacoline, arecaidine, guvacine, nitrosoguvacoline and arecoline‐d5 hydrobromide (spiked internal standard) in samples were analysed by UHPLC–MS/MS by a previously published method.^32^ A detailed description of chromatographic hardware, gradients of mobile phases and ionizing characteristics is provided in supporting information. MS/MS Instrument parameters for target alkaloids are presented in Table S1. A representative chromatographic separation of alkaloid and nitrosoguvacoline standards and detection by UHPLC–MS/MS is presented in Figure S1.

### 2.2 Preparation of aqueous extracts of LAN and AN

#### 2.2.1 Extraction into artificial enzymeless saliva (art‐Sal)

This process has been described in greater detail in a previous publication.^5^ On the day of processing L and green unripe AN were purchased from street vendors. L was trimmed of petiole and midrib and AN was trimmed of basal husk. Nine half leaves of L (7.4 g) and nine AN (36.5 g) were ground together for 2 min in a known volume of solution designed to ionically mimic human saliva. This artificial saliva (Art‐Sal) contained Na^+^—50 mM, K^+^—15 mM, Cl^—^20 mM, pH—7.2 buffered with 15‐mM Na di/mono phosphate.^33^, ^34^ This LAN pulp was pressed filtered, the exudate was centrifuged at 4°C at 4000 max xg for 2 min, and the volume of the supernatant collected was measured. Stock solution of LAN extract contained 0.608 g/ml AN and 0.124 g/ml L in Art‐Sal. The supernatant was stored on ice during aliquoting into Eppendorf tubes and immediately frozen at −20°C until use within 1 week. Aqueous extracts of 10 AN (37.7 g) (without L) in Art‐Sal were also prepared using the same procedure. Stock solution of AN was 0.598 g/ml Art‐Sal.

Manpower was not available to perform the in vitro chemical analysis as a double blinded experimental protocol. In order to control for the effect of time on experimental analysis, measurements of analytes in LAN were performed before and after each series of measurements to determine the effect of bases on alkaloids in LAN. Dose responses around the doses of bases used in human studies and a significant number of replications ensured accuracy of data.

#### 2.2.2 Experiment 1. Basal alkaloid levels in LAN or AN extracts

Defrosted stock samples of LAN and AN in Art‐Sal were vortexed and 100‐μl aliquot of LAN or AN was removed from the supernatant Eppendorf sample, which was returned to 4°C for subsequent use. The 100‐μl aliquot stock was vortexed for 3 min (length of chewing in human trials) at 21°C, centrifuged at 6000 max xg for 20 s. The supernatant was sampled and diluted 1000 times in 50% water/50% methanol and immediately injected into the UHPLC–MS/MS. Analytes in the stock solutions of LAN or AN were stable for 12 h at 4°C.

#### 2.2.3 Experiment 2a. Hydrolysis of LAN extract by suspension of Ca(OH)_2_ or Mg(OH)_2_

The 100 μl of LAN in Art‐Sal was reacted with suspensions of powdered Ca(OH)_2_ and Mg(OH)_2_. The final concentrations of Ca(OH)_2_ were 34 mg/g AN, 13.6 mg/g AN and 6.8 mg/g AN and Mg(OH)_2_ were 680 mg/g AN, 136 mg/g AN and 68 mg/g AN by vortexing for 3 min at 21°C before being processed as above for measurement by UHPLC–MS/MS. The pH of the final mixtures was not measured. Basal levels of analyte in aliquots of LAN stock were performed before and after each series of measurements with powdered Ca(OH)_2_ and Mg(OH)_2_, termed pre LAN and post LAN in graphs. Eight complete replicates were performed.

#### 2.2.4 Experiment 2b. Hydrolysis of AN extract by suspension of Ca(OH)_2_ and Mg(OH)_2_

The 100 μl of AN in Art‐Sal was reacted with suspensions of powdered Ca(OH)_2_ (13.6 mg/g AN) and Mg(OH)_2_ (136 mg/g AN) by vortexing for 3 min at 21°C before being processed as above for measurement by UHPLC–MS/MS. The pH of the final mixtures was not measured. Basal levels of analyte in aliquots of AN stock were determined before and after measurements with powdered Ca(OH)_2_ and Mg(OH)_2_, termed pre AN and post AN in graphs. Twelve complete replicates were performed.

### 2.3 Human experiments

This study follows the principles of the Declaration of Helsinki and was performed under the IRB ethics permit KMUH‐IRB‐20110270 issued by KMU.

#### 2.3.1 Experiment 3. Effect of chewing LAN, SBQ (LAN containing slaked lime) and novel SBQ (LAN containing Mg(OH)_2_) on physiological parameters of seated volunteers

##### Randomized pilot study

A pilot study was conducted on four male non‐smokers, occasional chewers of SBQ, aged 45 to 64 years, bwt 53–67 kg who gave informed, written consent and volunteered to chewed one each of LAN (5 g AN and 0.5‐g Piper leaf/65 kg bwt), SBQ (5‐g AN and 0.5‐g Piper leaf plus approximately 0.025‐g commercial slaked lime paste/65‐kg bwt) and novel SBQ (5‐g AN and 0.5‐g Piper leaf plus 0.5‐g Mg(OH)_2_ powder/65 kg bwt). Two subjects were recorded twice. A chewing time of 3 min was chosen as a compromise between the time needed to induce physiological effects (90 s) while attempting to minimize the muscular effect of chewing on the measurement of physiological parameters. This chewing time had been successfully employed in a previous SBQ characterization.^35^ This was not a double blinded experimental protocol. The order of presentation of the three chewing treatments was random. Volunteers were blind to the treatment being offered to chew but upon commencing chewing it was clear to the chewer which treatment was LAN without base. LAN without base tastes mildly astringent. The interval between chewing treatments was between 1 day and 1 week. Chewing protocol and measurement procedures for BP and HR were as per the main balanced study described in detail below. This study was used to optimize face temperature and eye photography. This experiment used a slightly lower dose of AN and LAN than was used in main study (below) and as such the HR and BP results of this study were only included in the linear regression between diastolic pressure and HR (Figure 8).

##### Balanced study

Eleven, non‐smoker, occasional chewers of SBQ, who chew 2–3 times annually, aged 29 to 64 years, 3 females and 8 males, bwt 53–97 kg gave informed, written consent and volunteered to chewed one each of LAN (6 g AN and 0.6 g Piper leaf/65 kg bwt), SBQ (6 g AN and 0.6 g Piper leaf plus approximately 0.03 g commercial slaked lime paste/65 kg bwt) and novel SBQ (6 g AN and 0.6 g Piper leaf plus 0.6 g Mg(OH)_2_ paste/65 kg bwt). The amount of Mg(OH)_2_ added was 100 mg/g AN wet weight. Mg(OH)_2_ was made as a paste with water in 2:3 w/w ratio and applied to the surface of the Piper leaf which was wrapped around the AN, as per SBQ presentation. SBQ, AN and L were purchase on the day of experiment from local retail outlets and the two SBQ were prepared for experiment immediately before chewing.

The randomized pilot study demonstrated that it was not practical to organize volunteers to complete three chewing treatments if chewing treatments were performed once per day. As a result, volunteers received the three chewing treatments in a single 4‐h period, starting at 12:30. In the first half an hour, subjects were weighted, seated, interviewed, eyes were photographed and face temp, HR and BP were measured in order to familiarize subjects with the experimental room and measurement protocol. This was not a double blinded experimental protocol. The order of presentation of the three chewing treatments was not random. Volunteers were blind to the treatment being offered to chew but upon commencing chewing it was clear to the chewer which treatment was LAN without alkalizing base. Preliminary experiments and randomized pilot study determined LAN without base tastes mildly astringent, the two SBS treatments tasted more palatable than LAN. In order to control for any effects of presentation order and time, five subjects chewed SBQ containing slaked lime first while six subjects chewed novel SBQ containing Mg(OH)_2_ first. In order to maximize the interval between chewing the two SBQ, LAN was always chewed as the middle treatment and a minimum of 2 h elapsed between chewing of the two SBQ treatments. Subjects were instructed to not be lazy and chew normally but with vigour. At least 1 h elapsed between chewing of SBQ and LAN. The half‐life of 5‐mg arecoline infused IV over 30 min period is 0.95 min.^36^ The time interval between chewing of SBQ and LAN was well in excess of the half‐life of arecoline infused into blood.

Physiological parameters were measured every 90 s for the duration of the experiment while subjects remained seated. Pre‐chewing baseline measurements were made for 6 min. While remaining seated, subjects chewed SBQ or LAN for 3 min. At 3 min, subjects ceased chewing and held quid and saliva in their mouth while measurements were made (approximately 30 s). While remaining seated, subjects spat out quid, rinsed their mouth with water (about 30 s) and sat still for the next 15 min while post treatment measurements were collected every 1.5 min. Measurements were conducted in an air conditioned room at 25°C with constant level of lighting. BP and HR was measured by a nurse using Omron Automatic Blood Pressure Monitor, Model HEM‐7210, Omron Healthcare Co. Kyoto, Japan with the blood pressure cuff transducer positioned at heart height for each volunteer. Left forehead surface skin temperature (about 1 cm above the left eyebrow) was measured using a skin contacting Omron Infrared Ear Thermometer, Model MC‐523, Omron Healthcare Co. Kyoto, Japan. Non‐contact IR face thermometers proved inaccurate once skin began to sweat. Pupils with scale marker, were photographed under conditions of constant lighting during baseline recordings *T* = −2.5 min and *T* = 0 min, at *T* = *F*, the onset of facial flushing, at *T* = 5 min coincident with maximal heart rate, at *T* = 10 min and *T* = 18 min. At *T* = *F* a series of photographs were taken every 10–15 s. Chewing LAN does not induce facial flushing and in this series *T* = *F* photo was taken at *T* = 3 min. Pupil and iris areas were analysed from photos using Fiji software.^37^ Subjects were instructed to self‐report if, and at what time, they experienced the onset of facial or thoracic flushing and sweating.

#### 2.3.2 Experiment 3b. Measurement of pH after mechanical mastication of three chewing treatments in human saliva for 3 min

Eight fresh LAN samples were weighed (wet weight [ww]). AN were split in quarters and LAN was dried for 24 h at 70°C. Eight healthy human volunteers 40–67 years old, five men, three women, all non‐smokers and non‐chewers, chewed a piece of silicon tube to stimulate production of saliva. Each volunteer provided 10‐ml saliva samples for each of the three chewing treatments. AN and L were trimmed to correct weight and pH of juice was measured from offcuts. Immediately upon producing each 10 ml, the saliva pH was measured and then the saliva was added to a mechanical chewer that masticated: (1) fresh LAN (0.5 g L + 5 g AN ww), (2) LAN + slaked lime (0.5 g L + 5 g AN ww + 0.025 g slaked lime − commercial SBQ) and (3) LAN + Mg(OH)_2_ (0.5 g L + 5 g AN ww + 0.5 g Mg(OH)_2_ powder) at 1 crush per 2 s. After 3 min the liquid was decanted from the quid and the pH was measured using universal indicator pH test paper (Advantec, Toyo Roshi Kaisha, Japan). pH values were a consensus between the saliva donating volunteer and PGO. When the reading was equivocal between two whole numbers the midpoint was recorded.

## 3 STATISTICS

The effect of concentrations of Ca(OH)_2_ and Mg(OH)_2_ on alkaloids in aqueous extracts of LAN or AN were analysed by repeated‐measures ANOVA with Tukey's Post hoc tests as corrections for multiple comparisons. Two tailed *P < 0.05, **P < 0.01, ***P < 0.001. Physiological parameters at the same time points for chewing LAN, LAN + slaked lime and LAN + Mg(OH)_2_ and pH after 3 min of mastication in human saliva were compared using repeated measures ANOVA with Tukey's Post hoc tests as corrections for multiple comparisons. Two tailed probabilities for LAN vs LAN + slaked lime. c *P* < 0.05, cc *P* < 0.01, ccc *P* < 0.001. Lan versus LAN + Mg(OH)_2._ m *P* < 0.05, mm *P* < 0.01, mmm *P* < 0.001. Paired *t*‐test was used to compare time of onset of facial flushing. All results are expressed as mean ± sem.

## 4 RESULTS

### 4.1 Experiments 1 and 2a. Hydrolysis of LAN extract by suspensions of Ca(OH)_2_ and Mg(OH)_2_

**FIGURE 1:**
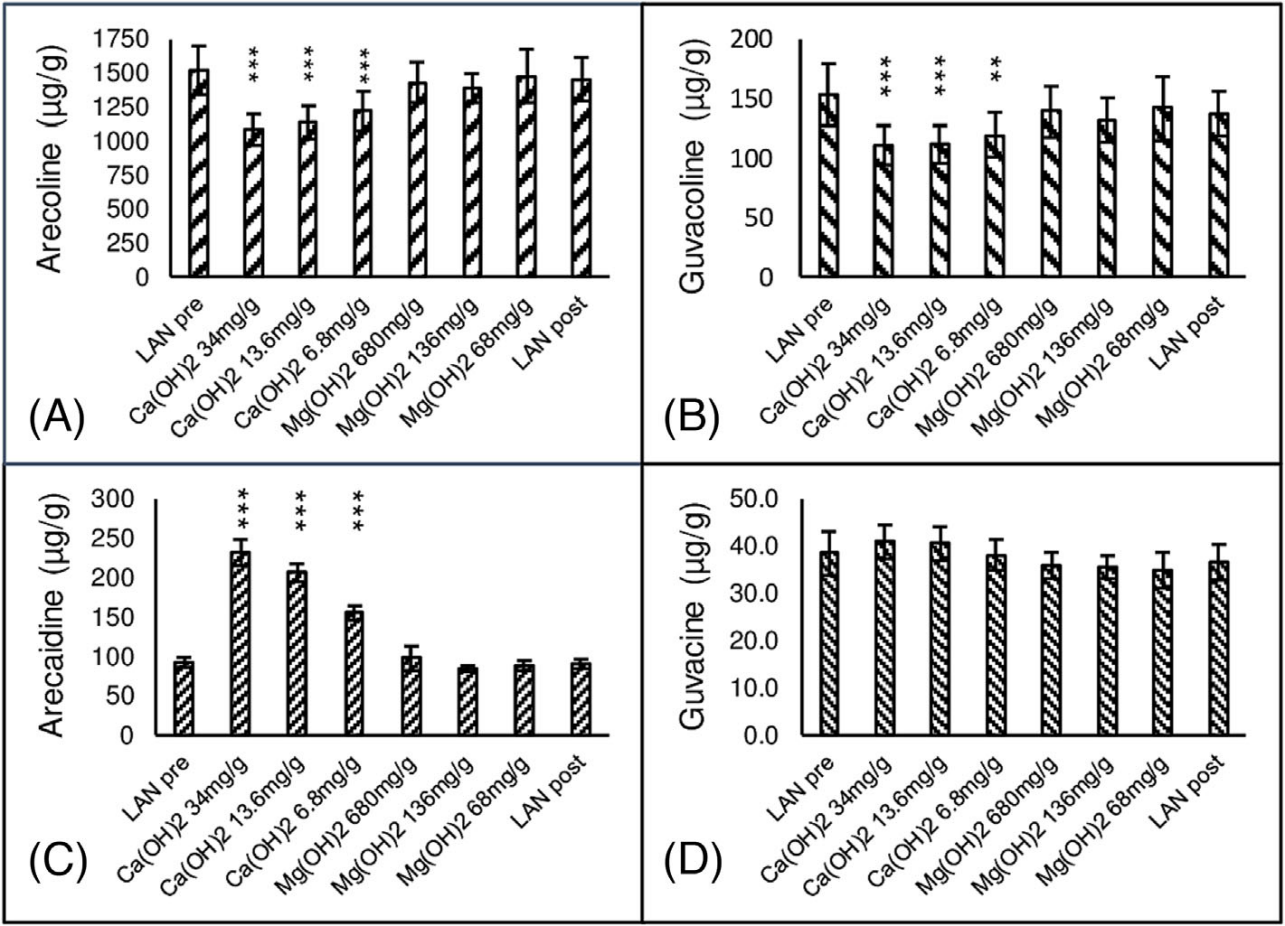
The effect of Ca(OH)_2_ and Mg(OH)_2_ on alkaloids in Areca nut and Piper leaf. The effect of 3‐min hydrolysis of aqueous extracts of Piper leaf combined with Areca nut (LAN) by Ca(OH)_2_ at doses of 34 mg/g AN, 13.6 mg/g AN and 6.8 mg/g AN and Mg(OH)_2_ at doses of 680 mg/g AN, 136 mg/g AN and 68 mg/g AN on esters, arecoline (A) and guvacoline (B), and carboxylic acid metabolites, arecaidine (C) and guvacine (D). Reaction conditions described in text. *N* = 8. Mean ± sem. LAN pre versus Other, ***P < 0.001, **P < 0.01.

Alkaloid concentrations in extracts of LAN are expressed as μg/g LAN wet weight. Alkaloid levels in LAN extracts were stable for 24 h at 4°C. Aqueous extracts of LAN had basal levels of arecoline 1507 ± 154 μg/g (*n* = 8), guvacoline 152 ± 22 μg/g (*n* = 8), arecaidine 94 ± 6 μg/g (*n* = 8) and guvacine 38 ± 4 μg/g (*n* = 8). The concentration of the esters, arecoline (*F*[7,44] = 14.7, *P* < 0.005) and guvacoline (*F*[7,44] = 8.8, *P* < 0.023) and the carboxylic acid, arecaidine (*F*[7,44] = 54, *P* < 0.0001) were significantly altered by incubation treatments. Guvacine concentration was not significantly altered by any treatment (*F*[7,44] = 3.9, *P* < 0.1). Figure 1 shows that incubation of LAN for 3 min with Ca(OH)_2_ at 34 mg/g AN, 13.6 mg/g AN and 6.8 mg/g AN dose dependently decreased the concentration of arecoline and guvacoline and dose dependently increased the concentration of arecaidine. Guvacine concentration was not significantly altered. Incubation of LAN for 3 min with Mg(OH)_2_ at 680 mg/g AN, 136 mg/g AN and 68 mg/g AN did not alter the concentration of the arecoline, guvacoline, arecaidine or guvacine. LAN extract was light yellow. Hydrolysis with Ca(OH)_2_ and Mg(OH)_2_ was accompanied by colour change to light orange which further darkened to dark red after about 30 min. Basal levels of all alkaloids in LAN extract did not change over the time course of the experiment as indicated by LAN pre and LAN post. Nitrosoguvacoline was not detected in any experiment.

#### 4.1.1 Experiment 2c. Hydrolysis of AN extract by suspension of Ca(OH)_2_ and Mg(OH)_2_

**FIGURE 2:**
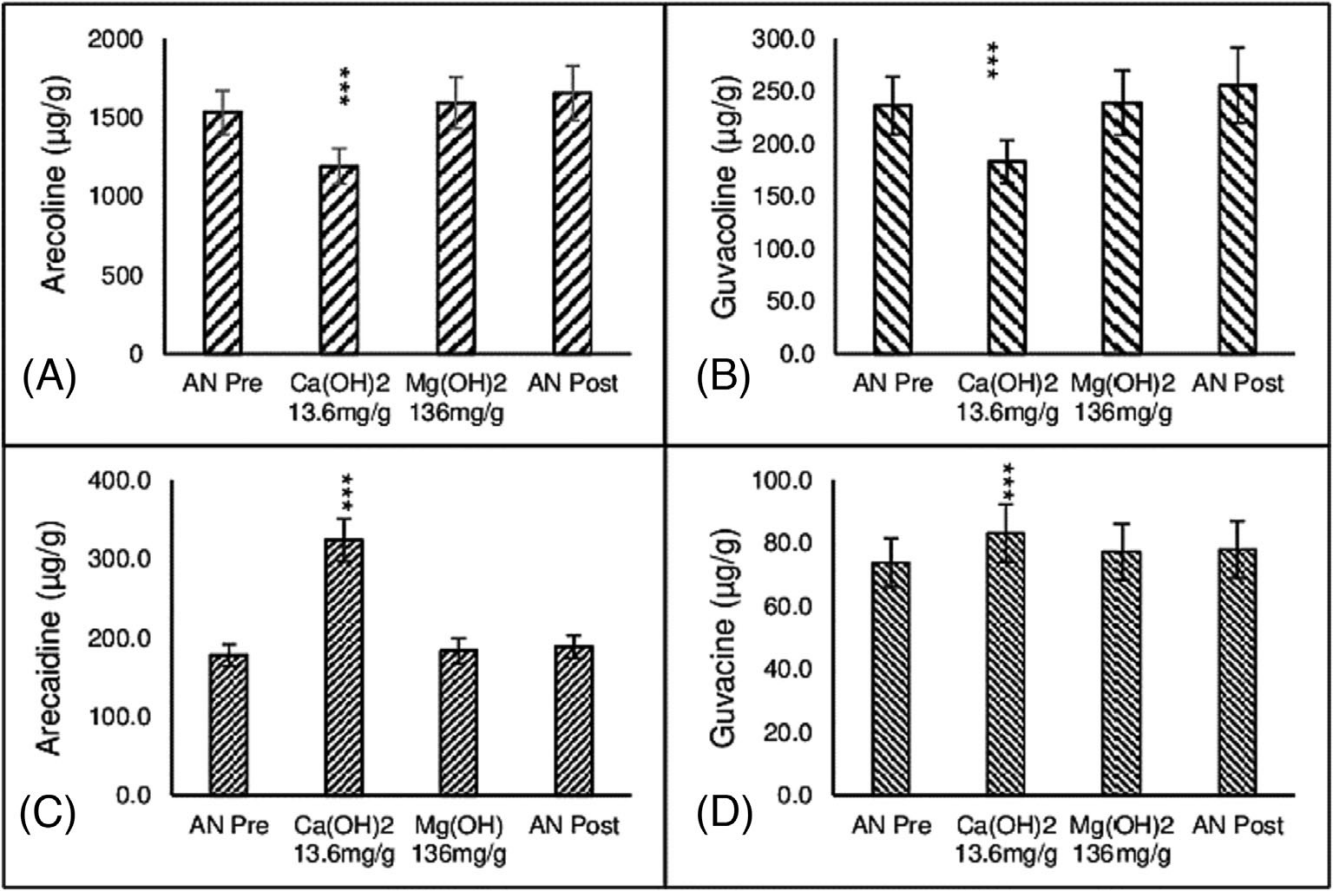
The effect of Ca(OH)_2_ and Mg(OH)_2_ on alkaloids in Areca nut. The effect of 3‐min hydrolysis of aqueous extracts of Areca Nut (AN) by Ca(OH)_2_ at 13.6 mg/g AN and Mg(OH)_2_ at 136 mg/g AN on esters, arecoline (A) and guvacoline (B), and carboxylic acid metabolites, arecaidine (C) and guvacine (D). Reaction conditions described in text. *N* = 12. Mean ± sem. AN Pre versus Other, ***P < 0.001.

Alkaloid concentrations in extracts of AN are expressed as μg/g AN wet weight. Aqueous extracts of AN had basal levels of arecoline 1532 ± 139 μg/g (*n* = 12), guvacoline 236 ± 27 μg/g (*n* = 12), arecaidine 178 ± 14 μg/g (*n* = 12) and guvacine 74 ± 8 μg/g (*n* = 12). The concentration of the esters, arecoline (*F*[3,33] = 27.2, *P* < 0.001), guvacoline (*F*[3,33] = 13.9, *P* < 0.001) and carboxylic acids, arecaidine (*F*[3,33] = 72, *P* < 0.0001) and guvacine (*F*[3,33] = 6.3, *P* < 0.01) were significantly influenced by incubation treatments. Figure 2 shows that incubation of AN extracts for 3 min with Ca(OH)_2_ at 13.6 mg/g AN decreased the concentration of arecoline and guvacoline and increased the concentration of arecaidine and guvacine. Incubation of AN for 3 min with Mg(OH)_2_ at 136 mg/g AN had no effect on the concentration of alkaloids. AN extract is clear. Hydrolysis was accompanied by colour change to light red which further darkened to dark red after about 30 min. Basal levels of all alkaloids in AN extract did not change over the time course of the experiment as indicated by AN pre and AN post. Nitrosoguvacoline was not detected in any experiment.

### 4.2 Human experiments

#### 4.2.1 Experiment 3. Effect of chewing LAN, SBQ (LAN containing slaked lime) and novel SBQ (LAN containing Mg(OH)_2_) on physiological parameters of seated volunteers

##### Subjective reporting of facial/thoracic flushing

No subject reported facial or thoracic flushing to chewing LAN. Every subject reported facial and upper thoracic (face, chest and upper back and more rarely forearms and hands) flushing to chewing SBQ and novel SBQ. The onset of flushing ranged between 30 to 149 s post the onset of chewing. Mean onset of flushing for LAN + slaked lime was 99 ± 9 sec and for LAN + Mg(OH)_2_ was 109 ± 14 sec (*n* = 11) (*t*(10) = −1.0, *P* = 0.3). Thoracic flushing and sweating was frequently, but not always, accompanied by a feeling of dizziness. Flushing and dizziness frequently persisted until T = 7 min, about 3 min after subjects had finished chewing SBQ. Subjects indicated that flushing to SBQ was invariably, but not always, stronger than novel SBQ. SBQ tasted sweeter than novel SBQ, both of which tasted better than LAN. Mental effects were noted during the flushing but no consensus of feeling other than head tightness was described.

##### Forehead temperature

**FIGURE 3:**
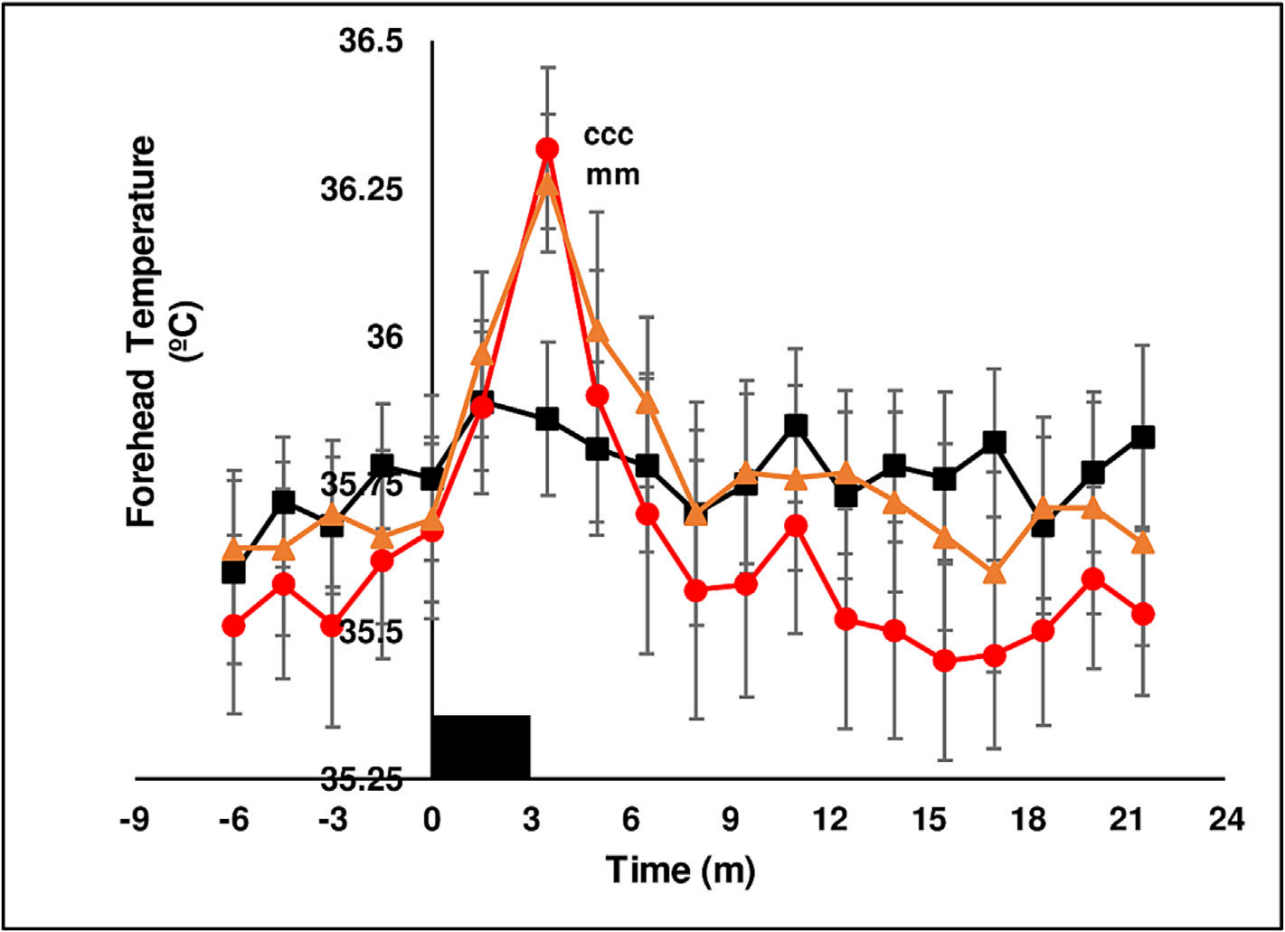
The effect of three chewing treatments on forehead temperature. The effect of chewing LAN (black solid squares), LAN + slaked lime (red solid circles) or LAN + Mg(OH)_2_ (orange solid triangles) for 3 min (solid black rectangle) on forehead temperature (°C) in 10 seated subjects. Mean ± sem. LAN versus LAN + slaked lime, ccc *P* < 0.001. LAN versus LAN + Mg(OH)_2_, mm *P* < 0.01.

Basal forehead temperature (35.7 ± 0.03°C) was not different between chewing treatments. Forehead temperature was not increased by chewing LAN. Forehead temperature was maximal at 3.5 min after the onset of chewing SBQ. The maximal increase in forehead temperature for chewing LAN + slaked lime was 0.74 ± 0.08°C (*n* = 10) and for chewing LAN + Mg(OH)_2_ was 0.59 ± 0.04°C (*n* = 10). Forehead temperature returned to basal levels once sweating began. Data from one subject was lost. An increase in face temperature after chewing SBQ is consistent with previous reports.^35^, ^38^ The time course of the temperature response to chewing treatments is presented in Figure 3.

##### Hemodynamic parameters

**FIGURE 4:**
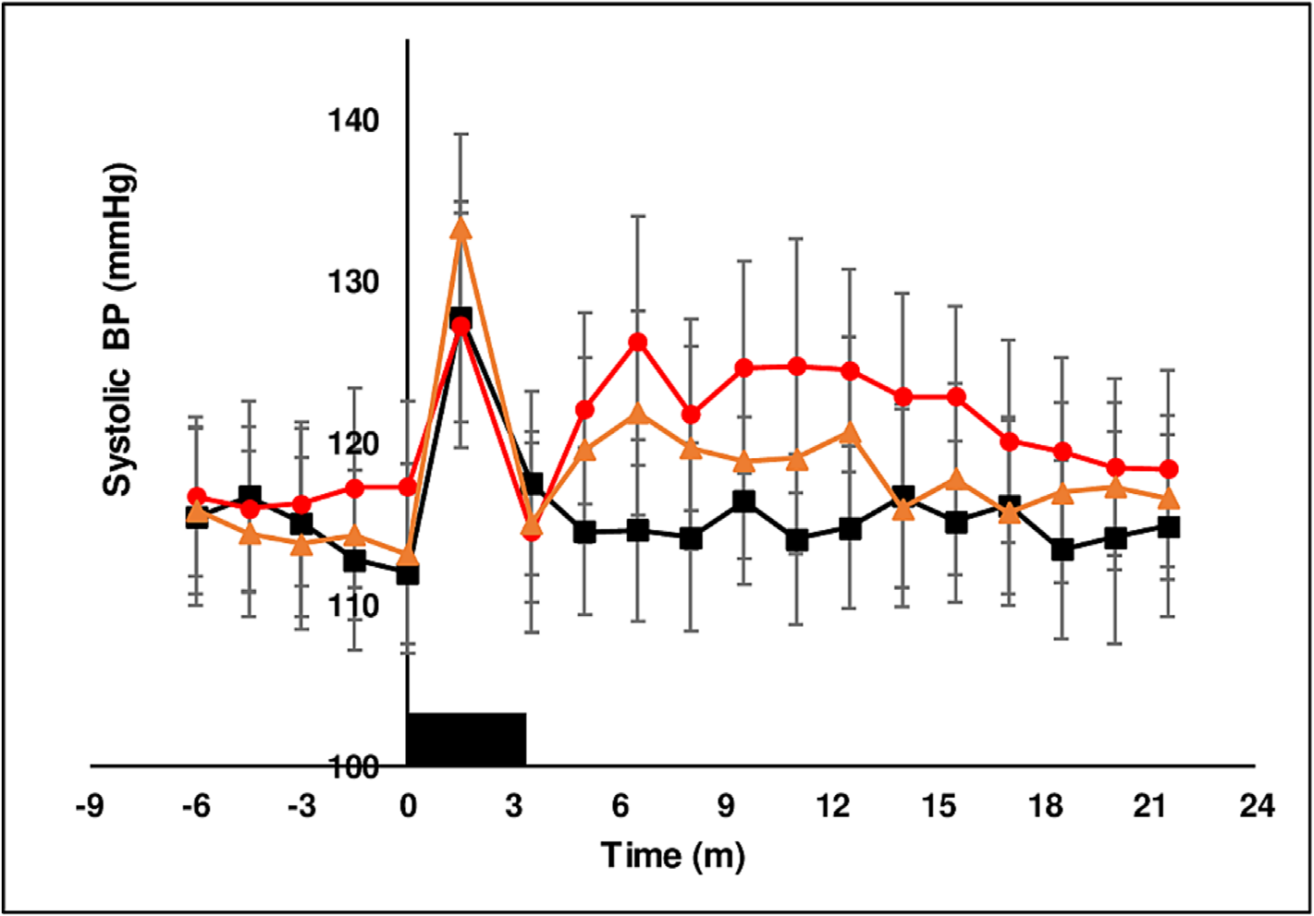
The effect of three chewing treatments on systolic BP. The effect of chewing LAN (black solid squares), LAN + slaked lime (red solid circles) or LAN + Mg(OH)_2_ (orange solid triangles) for 3 min (solid black rectangle) on systolic BP in 11 seated subjects. Mean ± sem.

**FIGURE 5:**
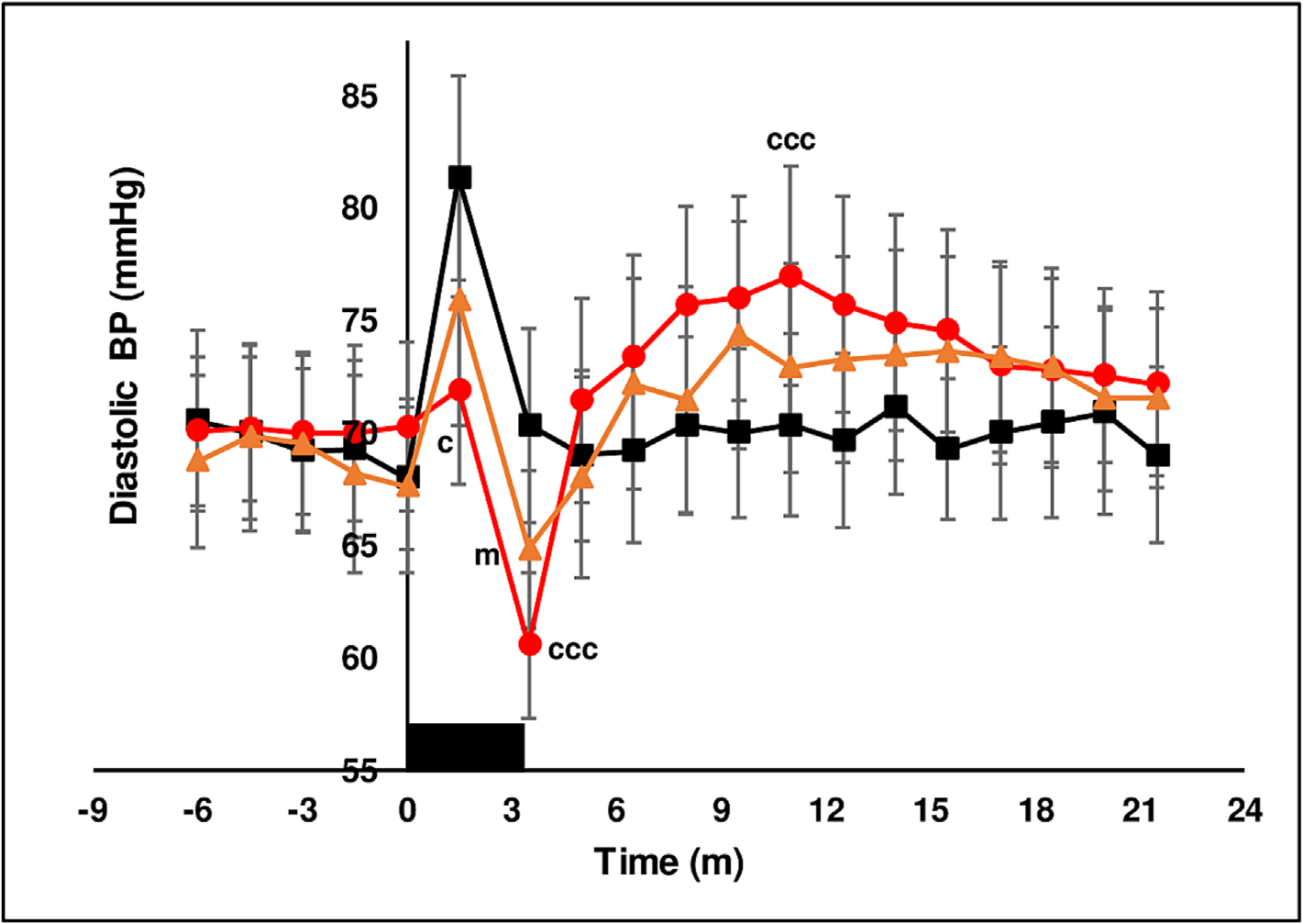
The effect of three chewing treatments on diastolic BP. The effect of chewing LAN (black solid squares), LAN + slaked lime (red solid circles) or LAN + Mg(OH)_2_ (orange solid triangles) for 3 min (solid black rectangle) on diastolic BP in 11 seated subjects. Mean ± sem. LAN + slaked lime versus LAN, ccc *P* < 0.001, c *P* < 0.05. LAN + Mg(OH)_2_ versus LAN, m *P* < 0.05.

**FIGURE 6:**
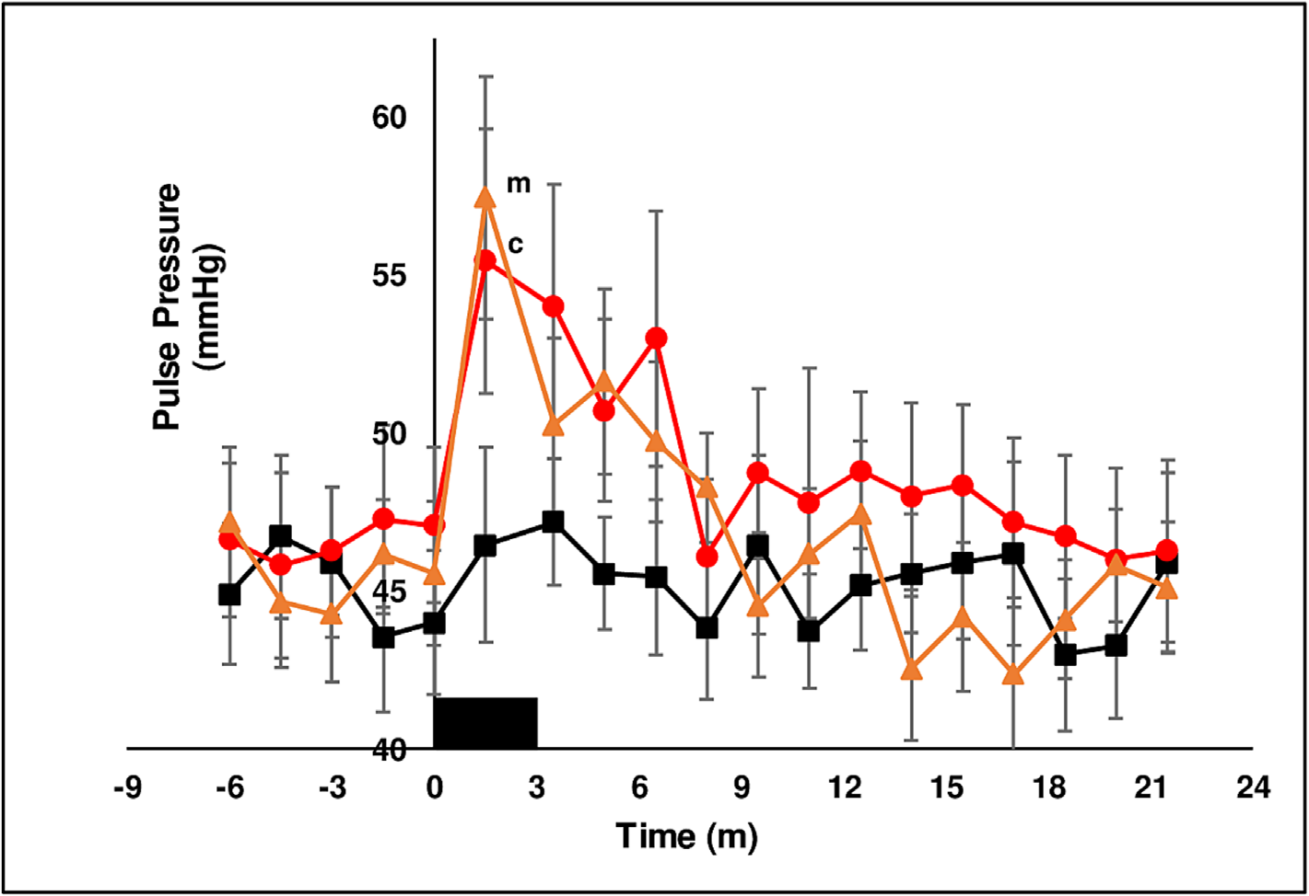
The effect of three chewing treatments on pulse pressure. The effect of chewing LAN (black solid squares), LAN + slaked lime (red solid circles) or LAN + Mg(OH)_2_ (orange solid triangles) for 3 min (solid black rectangle) on Pulse Pressure (mmHg) in 11 seated subjects. Mean ± sem. LAN versus LAN + slaked lime, c *P* < 0.05. LAN versus LAN + Mg(OH)_2_, m *P* < 0.05.

**FIGURE 7:**
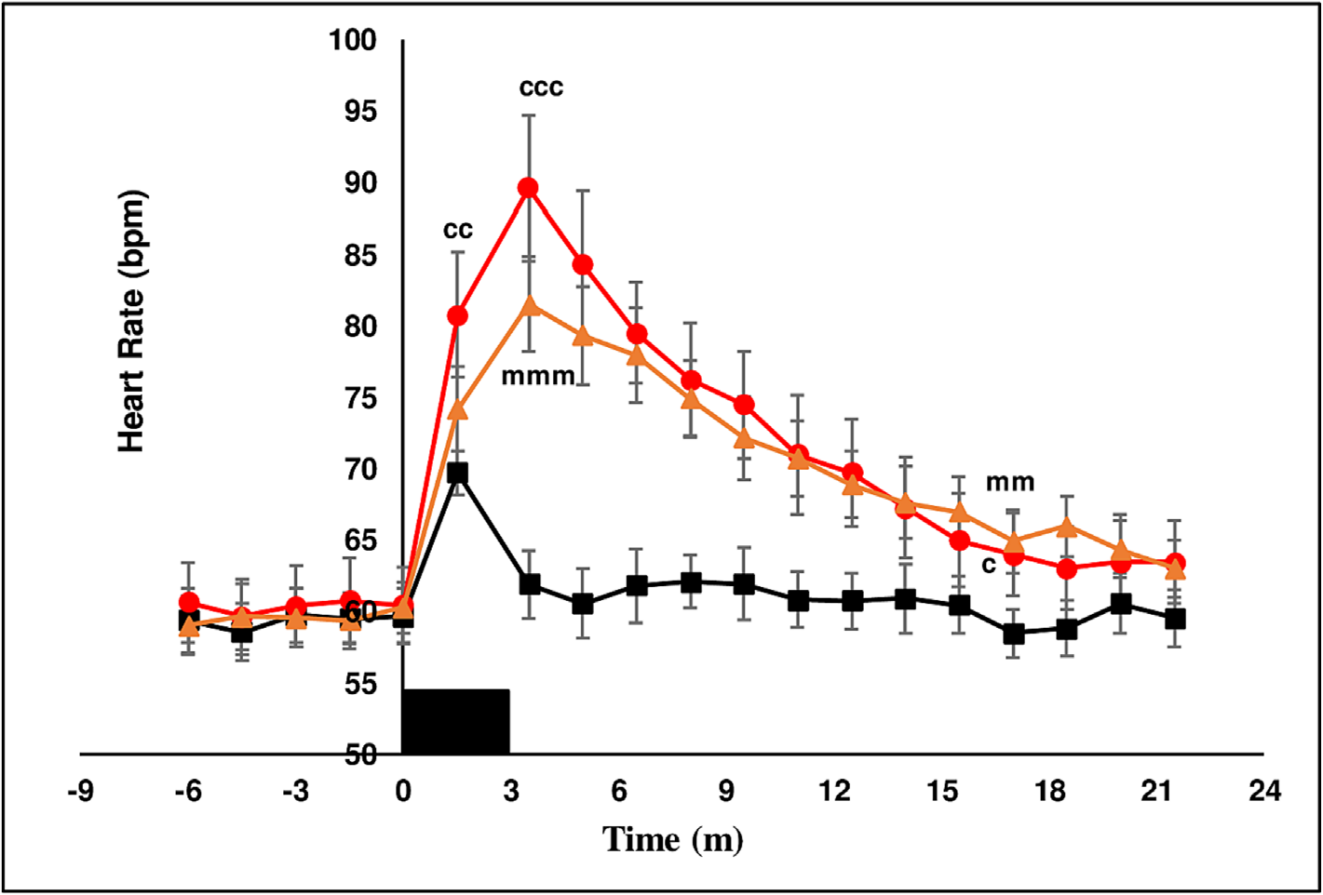
The effect of three chewing treatments on heart rate. The effect of chewing LAN (black solid squares), LAN + slaked lime (red solid circles) or LAN + Mg(OH)_2_ (orange solid triangles) for 3 min (solid black rectangle) on heart rate (bpm) in 11 seated subjects. Mean ± sem. LAN versus LAN + slaked lime, c *P* < 0.05, cc *P* < 0.01, ccc *P* < 0.001. LAN versus LAN + Mg(OH)_2_, m *P* < 0.05, mm *P* < 0.01, mmm *P* < 0.001.

Basal systolic (115 ± 0.5 mmHg), diastolic pressure (69.4 ± 0.3 mmHg) and heart rate (59.8 ± 0.2 bpm) were not different between treatment chewing groups in the baseline period prior to chewing. The hemodynamic response to the muscular exercise of chewing is to increase BP and HR as was observed with chewing LAN (see below). The experimental design minimized this effect by stopping chewing at 3 min and measuring the effect of chewing on hemodynamic parameters in the interval from 3 to 21.5 min. Chewing LAN increased BP and HR above baseline at *T* = 1.5 min. BP and HR returned to basal levels when chewing ceased at 3 min and remained unchanged for the duration of the measurement period. From *T* = 1.5 min to *T* = 3.5 min chewing both SBQ induced a diminished increase in systolic pressure (Figure 4), decreased diastolic pressure (Figure 5), briefly increased pulse pressure (Figure 6) and increased HR (Figure 7) coincident with the onset of thoracic flushing. Dizziness and cold sweats were coincident with decrease in diastolic BP. At *T* = 3.5 min systolic BP was normal relative to baseline, while HR was maximally increased, diastolic pressure was decreased and forehead temperature was maximal, collectively indicative of a thoracic vessel vasodilation and an effective pseudo‐hypovolemia. In the interval after *T* = 5 min, HR was substantially elevated while systolic and diastolic BP achieved a significant increase at only a single time point. After chewing both LAN + slaked lime and LAN + Mg(OH)_2_ HR was maximal at *T* = 5 min after the onset of chewing and had returned to basal levels by *T* = 18 min. An increase in HR after chewing orthodox SBQ is consistent with previous reports.^35^, ^39^

**FIGURE 8:**
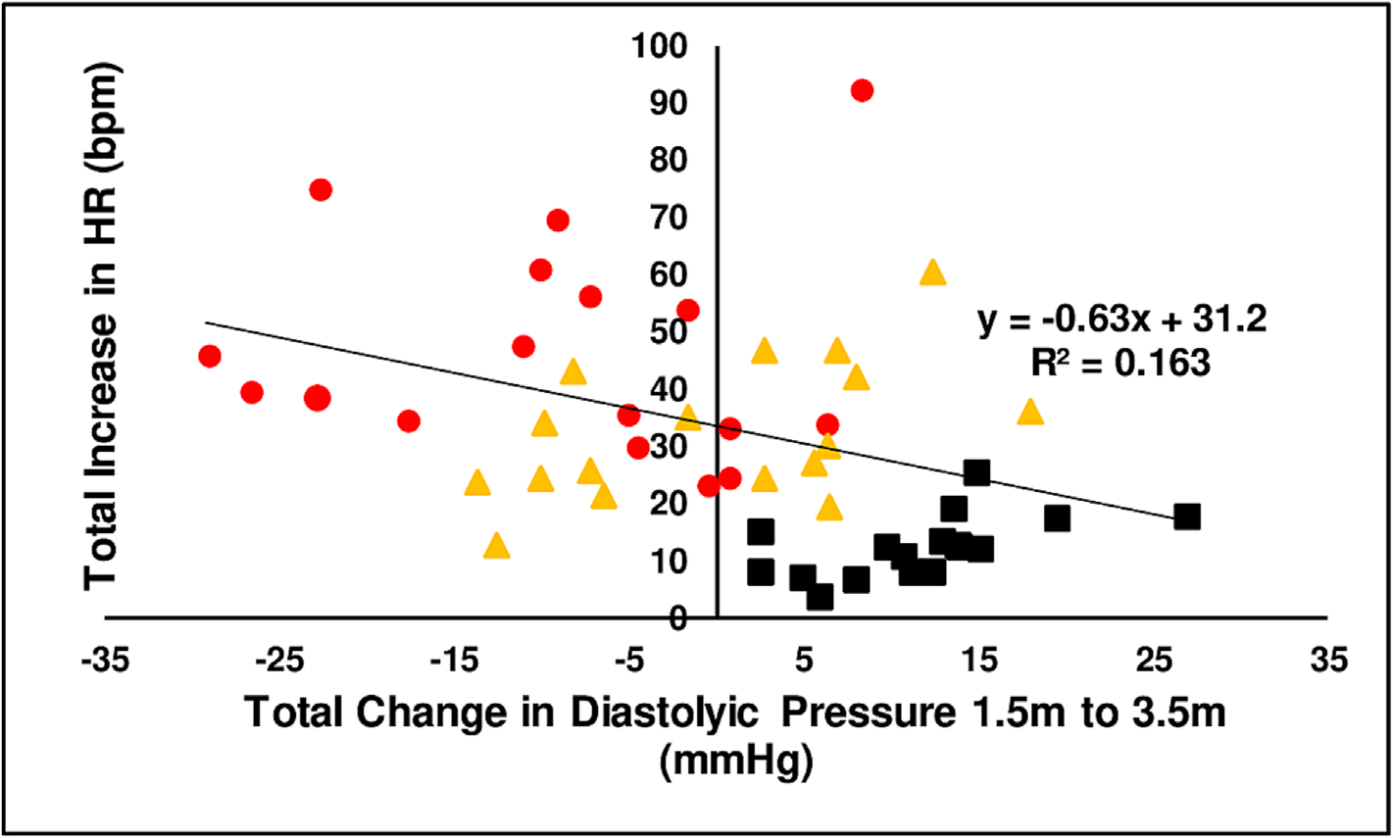
The effect of three chewing treatments on heart rate and diastolic pressure. The correlation between cumulative increase in HR and cumulative decrease in diastolic pressure recording from 1.5 to 3.5 min after chewing LAN (black solid squares), LAN + slaked lime (red solid circles) or LAN + Mg(OH)_2_ (orange solid triangles) for 3 min in 17 people.

The results from six pilot studies where included in an analysis of the relationship between the cumulative change in diastolic BP and HR in the time interval from *T* = 1.5 min and *T* = 3.5 min relative to basal pre chewing values for LAN, LAN + slaked lime and LAN + Mg(OH)_2_ (*n* = 17). (*F*[1,49] = 9.6, *P* = 0.003). Figure 8 shows the relationship and linear regression of these parameters.

##### Pupil parameters

**FIGURE 9:**
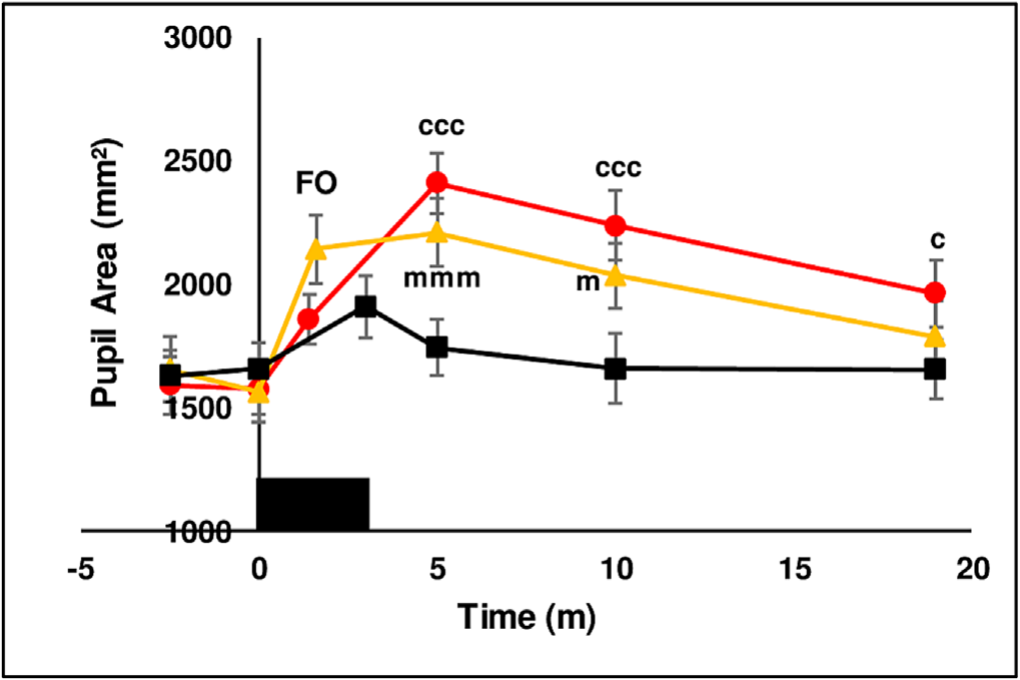
The effect of three chewing treatments on pupil area. The effect of chewing LAN (black solid squares), LAN + slaked lime (red solid circles) or LAN + Mg(OH)_2_ (orange solid triangles) for 3 min (solid black rectangle) on pupil area (mm^2^) in five seated subjects (10 pupils). Mean ± sem. FO = flush onset. LAN versus LAN + slaked lime, ccc *P* < 0.001, c *P* < 0.05, LAN versus LAN + Mg(OH)_2_, mmm *P* < 0.001, m *P* > 0.05.

**FIGURE 10:**
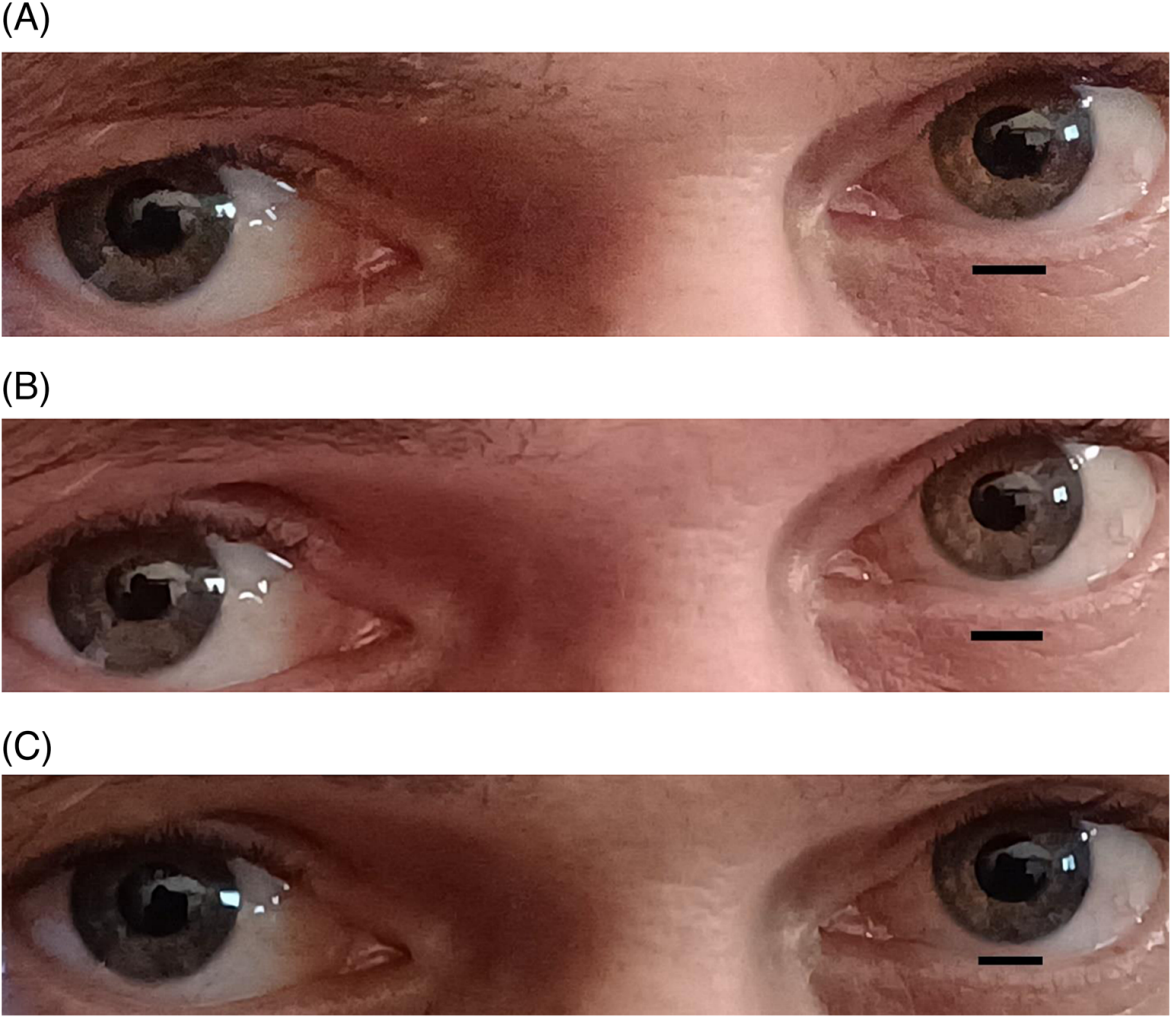
Representative effect of three chewing treatments on pupil area. Representative photographs of eyes of one subject at *T* = 5 min chewing (A) LAN + Mg(OH)_2_, (B) LAN, and (C) LAN + slaked Lime. Scale bar in each photo is 80 mm in length.

Pupils were photographed in 11 subjects however quantitative changes could only be measured from five subjects with light coloured irises. Figure 9 shows that chewing LAN transiently increased pupil diameter at *T* = 3.5 min. Chewing of both SBQ treatments dilated pupils with maximal increase recorded 2 min after chewing ceased (*T* = 5 min). In some subjects, pupils had not dilated at the onset of perceived flushing (FO) but increased as photographs were being taken over the next 30 s. The difference in magnitude of pupil dilation at FO between the two SBQ probably reflects a difference in accuracy of perceiving the onset of flushing. Qualitative visual ranking of photographs showed that pupils of all subjects dilated after chewing SBQ. Pupils were never observed to contract during chewing of SBQ or LAN. Figure 10 shows the effect of the three chewing treatments at *T* = 5 min on pupil area is shown in a representative subject.

#### 4.2.2 Experiment 3b. Measurement of pH after mechanical mastication of three chewing treatments in human saliva for 3 min

Prior to drying, LAN was 52.3 g ww. After drying for 12 h at 70°C the weight of LAN was 9.1 g. The moisture content of LAN was 83%. There was considerable variation between the eight volunteers in the time taken to produce 10 ml stimulated saliva samples. A range between 3 min to 10 min was recorded. The pH of human saliva samples, AN juice and Leaf juice was measured prior to each of the three chewing treatments for each subject (*n* = 24). The mean of the three values of each of human saliva samples, AN juice and Leaf juice pH (*n* = 8) was used for statistical analysis. Table 1 shows that human saliva was pH 7.3. The pH of human saliva was significantly different from pH of AN juice, Leaf juice and all chewing treatments (*F*[5,35] = 437, *P* = 0.0000). Fresh AN juice was pH 4.1. Fresh Leaf juice was pH 4.2. After 3 min of mechanical chewing of LAN in 10 ml of human saliva the pH of pulp solution was pH 5.4. After 3 min of mechanical chewing of LAN + slaked lime in 10 ml of human saliva the pH of pulp solution was pH 10.8. After 3 min of mechanical chewing of LAN + Mg(OH)_2_ in 10 ml of human saliva the pH of pulp solution was pH 9.2. In both SBQ, a colour change in quid was noticeable at about 1 min after chewing commenced. The ratio of total water content of LAN to saliva used in these experiments was 1:2.5 respectively.

**TABLE 1:** The effect on pH of 3 min of mechanical chewing of LAN, LAN + slaked lime and LAN + Mg(OH)_2_ in 10 ml of human saliva from eight volunteers.

			pH after 3 min of mechanical chewing		
Stimulated human saliva pH.	AN juice pH.	Leaf juice pH.	LAN (5.5 g ww) + 10 ml saliva pH.	LAN (5.5 g ww) + 10 ml saliva + slaked lime (5 mg/g AN) pH.	LAN (5.5 g ww) + 10 ml saliva + Mg(OH)_2_ (100 mg/g AN) pH.
Mean (range)[n]	Mean (range)[n]	Mean (range)[n]	Mean (range)[n]	Mean (range)[n]	Mean (range)[n]
7.3 (7–8) [8]	4.1*** (4‐5) [8]	4.2*** (4‐5) [8]	5.3*** (5‐6) [8]	10.8 *** (10–11) [8]	9.2 *** (8.5–10) [8]
milky	clear	clear	green	red	orange/red

*Note*: Stimulated human saliva versus others.

*** *P* < 0.001.

## 5 DISCUSSION

In these experiments we expanded the physiological parameters previously measured and confirm our previous finding that chewing LAN induced small, transient increases in HR and face temperature^5^ of a magnitude similar to chewing gum in a similar experimental protocol.^35^ The physiological effects of systemically administered arecoline are complex to interpret because, being able to cross the blood brain barrier, it has concurrent peripheral and central actions. Human experiments on Alzheimer's patients utilizing IV arecoline to treat memory loss required peripheral, non CNS penetrating, muscarinic blockade to limit sweating, nausea and vomiting induced by systemic arecoline. After blockade, subcutaneous or intravenous infusions of 2–8 mg of arecoline induce short term increase in heart rate, blood pressure and longer term reduction in core temperature. Cognitive improvements but also nausea were observed.^20^, ^40^, ^41^ In the current experiments and our previous experiments^5^ none of these peripheral or central effects were observed when chewing LAN which contained 8‐9 mg of arecoline. The discrepancy between earlier IV studies and this chewing study indicates that the high levels of arecoline in LAN are not absorbed across the oral membranes to influence physiology.

Fresh extracts of unripe AN juice were pH 4.1, consistent with previous measurements.^4^ Fresh extracts of Piper leaf were pH 4.2. The pH of saliva spat out after the three in vivo chewing treatments was not measured, but in vitro simulation of masticating LAN in human saliva for 3 min showed that stimulated saliva has a pH of about 7.3, consistent with previous measurements,^4^, ^33^, ^34^ but demonstrated only a weak buffering capacity, consistent with previous measurements.^33^ At the volume ratios employed of LAN 1: Saliva 2.5, saliva raised the acidic pH of LAN extracts by 1 pH unit but was unable to buffer the LAN solution to physiological levels. Since the pKa of arecoline is 7.2–7.6^4^, ^42^ and the simulated oral pH after 3 min of chewing LAN was 5.3, the Henderson‐Hasselbalch equation predicts that at this time most of the arecoline will be in an ionized form and will have low passive permeability to cell membranes.^21^ This interpretation is consistent with our observations of no obvious activation of the autonomic system after 3 min of chewing LAN. It is highly probable that arecoline, and other polar phytochemicals derived from LAN, stay in the saliva and will be spat out or swallowed and digested in the stomach. It is conceivable that an individual's salivary flow rate and buffering capacity could have considerable influence on both the short and long term outcome of chewing SBQ.

Reconciling the disparity between the lack of physiological effect of chewing LAN and the animal and cell culture literature demonstrating that extracts of AN or arecoline are cytopathogenic is crucial. The pKa of arecoline is slightly basic, near neutral and as such small changes in pH of incubation mediums can have significant impacts on drug absorption, distribution, metabolism, excretion/elimination and toxicity.^21^ Re‐examination of past toxicology studies to determine if experiments reporting carcinogenic effects utilized an extracellular incubation milieu that mimicked the oral acidic pH of AN and LAN extracts in saliva or employed a more cell friendly physiological, near neutral pH, has the potential to reveal the origin of this discrepancy. The IARC 2004 monograph found no direct evidence for carcinogenicity from chewing AN but did find evidence of oral submucosa fibrosis, a precancerous condition in humans and chose to categorize AN as a type 1 carcinogen. However, the epidemiological data summarized in the 2004 monograph as AN is contestable while the animal and cell culture data for the effects of AN are not robust and it is difficult to envisage any mechanism whereby these earlier findings are compatible with the absence of the physiological effects of arecoline after chewing LAN and the co‐incident theoretical evidence of the existence of predominantly polar species of low lipophilia.

Could experiments be performed where LAN is added as a culturally appropriate placebo supplement to alleviate cravings as part of a contingency management plan in SBQ SUD quitting programs that currently focus therapy around abstinence? When viewed in the light of health policies based upon the findings of the IARC 2004 monograph, such a treatment initiative would definitely be unacceptable because instead of abstinence the proposed new treatment is promoting the use of a potential carcinogenic substance. However, in theory, the inclusion of LAN as a placebo has considerable merit. LAN is a culturally appropriate placebo, easy to obtain, geographic distribution is as per SBQ, requires minimal re‐education, requires no training to use and is not costly in professional human resources to administer. The inclusion of a placebo supplement has good potential to increase the completion and efficacy of quitting programs which will decrease the chewers cumulative exposure to carcinogens thereby decreasing the statistical risk of ill health or cancer.^22^, ^28^, ^43^ Philosophically, the indirect cost of any failed attempt to quit is the direct promotion of the use of a known carcinogenic substance (SBQ containing lime). If abstinence treatment is a less successful quitting strategy than abstinence plus placebo treatment, then abstinence treatment is the greater promoter of the use a known carcinogenic substance. Chewing LAN with no slaked lime instead of SBQ containing slaked lime must be chemically less destructive on the oral mucosa^44^ and this is definitely a form of decreasing harm to the chewer (Discussed in greater detail below). Finally, if a placebo has a rational potential to improve efficacy of a quitting attempt, should this putative benefit not be evaluated?

This paper presents the most comprehensive description of the time course of physiological changes induced by chewing SBQ. In these experiments we show for the first time that the physiological profile of SBQ containing LAN + slaked lime and novel SBQ containing LAN + Mg(OH)_2_ are essentially equivalent. Based upon the parameters measured, the initial physiological response to compounds in LAN by slaked lime or Mg(OH)_2_ appears to be the induction of rapid dilation of thoracic and facial vasculature inducing a transient hypotension equivalent to a thoracic pseudo‐hypovolemia. Given the temporal limits of the non‐invasive procedures employed, the onset of diastolic hypotension and increased pulse pressure to LAN + slaked lime and LAN + Mg(OH)_2_, correlate well with the time of self‐reported onset of facial flushing, sweating and increased face temperature induced by chewing both SBQ. A clear relationship between magnitude of increase in HR and decrease in diastolic pressure was recorded over the few minutes around the onset of facial and thoracic flushing induced by chewing the two SBS. Interestingly, pupils did not constrict at the onset of hypotension and flushing but dilated quickly after onset of flushing. This response profile suggests that SBQ induced hypotension is a thoracic vascular specific effect and not a response of global thoracic activation of parasympathetic activity. A compensatory homeostatic stimulation of sympathetic activity producing pronounced tachycardia, slight elevation of diastolic BP and pupil dilation is quickly induced in response to the transient hypotension/pseudo‐hypovolemia. The long duration of the tachycardia and pupil dilation in the absence of an elevation of BP, suggests that the hypotensive action of compounds from LAN is long acting. However, thoracic flushing and dizziness are short lasting, not being evident 3 min after chewing has stopped. This temporal inconsistency is interpreted as evidence that in the early stage of SBQ intoxication, the activation of sympathetic mechanisms results from homeostasis but the more protracted sympathetic activation results from the central stimulant action of compounds in LAN or produced by hydrolysis of LAN. No attempt was made to access mental effects of these two SBQ treatments as currently there is no test for quantifying SBQ intoxication. A brief mental confusion and head tightening was noticeable at 2–4 min after chewing commenced in both SBQ.

Our in vitro analysis, designed to simulate oral conditions of chewing, demonstrated that chewing LAN or AN with slaked lime decreased the levels of muscarinic esters, arecoline and guvacoline via hydrolysis and increased the levels of the carboxylic GABAergic uptake inhibitor metabolites, arecaidine consistent with previous studies.^4^, ^5^, ^45^, ^46^, ^47^, ^48^ Guvacine levels were increased in assays of AN and not significantly increased in assays of LAN. Other functionally active unknown compounds may have been produced by hydrolysis although the mutagenic compound, nitrosoguvacoline, was not detected. In the current experiments, the physiological profile of SBQ containing LAN + slaked lime and the novel SBQ containing LAN + Mg(OH)_2_ are indistinguishable, yet analytically, Mg(OH)_2_ in vitro did not hydrolyze the muscarinic esters in aqueous extracts of LAN and AN. This is consistent with the fact that Mg(OH)_2_ is a weak base (pH = 10.1) of very poor solubility^49^ and the recent demonstration that the hydrolysis of arecoline to arecaidine is a pH sensitive process that proceeds above pH 10.^4^ Although Mg(OH)_2_ does not hydrolyze the alkaloids in extracts of LAN and AN, it is chemically active as it induced a green to orange colour change in the extract.

Phytochemicals in LAN do not induce stimulatory physiology when chewed however the same phytochemicals when chewed with slaked lime or Mg(OH)_2_ induce potent parasympathetic followed by sympathetic activation. In vitro, human saliva buffered the pH of the alkaline bases by about 1 pH unit and the simulated oral pH of masticating LAN + slaked lime was 10.8. The simulated oral pH of masticating LAN + Mg(OH)_2_ was 9.2. Alkaloids are basic with pKa specific to their chemical structure. On the basis of the pH‐partition hypothesis, chewing LAN with a strong alkali, such as slaked lime, or mid strength alkali, such as Mg(OH)_2_, increases the pH of the mouth milieu, which increases the conversion of ionized alkaloids to unbound, unionized alkaloids thereby increasing the passive permeability of these now nonpolar drugs across the oral cell membranes.^21^ Concurrently, by virtue of its higher pH, slaked lime but not Mg(OH)_2_, metabolize muscarinic esters in LAN to GABAergic uptake inhibitors. In a previous study of chewing LAN and SBQ containing slaked lime, physiological activation after chewing correlated negatively with in vitro levels of muscarinic esters and correlate positively with the levels of GABAergic uptake inhibitors^5^ but this correlation has now been shown to be invalid after the inclusion of the Mg(OH)_2_ treatment. The millennium old practice of adding slaked lime to LAN to facilitate the absorbance of compounds and metabolites in SBQ into cells of the mouth to induce physiological and psychological effects was a remarkable confluence of historical ingenuity and resources.^50^ Our experiments demonstrate for the first time that other weaker bases can be used to induce the same physiological effects and that the alkaline strength of slaked lime is probably in excess of chemical need. The obvious candidate to mediate the autonomic aspects of the physiological response is arecoline. The compound mediating the long duration central sympathetic stimulation observed after the onset of the autonomic response is unknown.

It is well recognized that prolonged chewing of SBQ containing slaked lime is carcinogenic and harmful to many organs of the body.^23^, ^24^ However, the aetiology of pathology is poorly understood, but considered a combination of the direct action of extremely unphysiological alkaline pH of slaked lime on mucosal cells^44^ and the engendered chemistries of overly energetic hydrolysis of compounds in LAN and cells of the mouth, possibly inappropriate free radical production^51^ and the actions of intracellular arecoline^52^, ^53^ combining to over stimulate activation of cellular pathways of inflammation.^6^, ^54^

Could SBQ containing Mg(OH)_2_ be used as culturally appropriate replacement therapy for SBQ containing slaked lime for chewers with SUD who are unable to abstain from chewing, if it could be demonstrated that chewing SBQ containing Mg(OH)_2_ was reducing harm to the chewer relative to chewing SBQ containing slaked lime? Adherence to current health policies based upon the IARC 2004 monograph would conclude that this was definitely unacceptable because instead of abstinence the proposed replacement therapy is promoting the use of a potential carcinogenic substance. However, in theory, SBQ containing Mg(OH)_2_ replacement therapy has considerable merit as it has numerous theoretical, but as yet unproven benefits, over chewing SBQ containing slaked lime.

Given the etiological uncertainties that generate pathology associated with chewing SBQ containing LAN + slaked lime, combined with the fact that this is the first demonstration that chewing SBQ with bases other than slaked lime can stimulate a physiological response, there is no direct experimental proof that chewing SBQ containing LAN + Mg(OH)_2_ is less harmful than chewing of SBQ containing LAN + slaked lime. However, much of the basis of cytopathological chemistry of SBQ is at its essence, dependent upon the high pH and solubility of the components in slaked lime. Since Mg(OH)_2_ has lower pH, lower solubility and is less reactive than the components of slaked lime, as exemplified by the measured decrease in ability to hydrolyze alkaloid compounds in LAN, it theoretically has a decreased ability to induce cytopathology, while Mg^2+^ the ion, has some cyto‐protective properties, listed below. In this context it should be acknowledged that Mg(OH)_2_ is FDA approved for human consumption^31^ and the principal compound in Milk of Magnesia that is an over the counter medication used as antacid to treat occasional heart burn and as a laxative to treat occasional constipation.^49^ The long term effects of Mg(OH)_2_ consumption are unknown because it is not taken as a long term medication but it would be prudent to acknowledge the potential for diarrhoea. It should also be considered that when chewing SBQ, the juice is invariably spat out so the dose consumed is considerably less than the dose employed. It should also be acknowledged that the compounds that make up slaked lime, Ca(OH)_2,_ ^55^ NaOH^56^ and KOH^57^ are all listed by the Centers for Disease Control as causing corrosive damaging to eyes, skin, mucous membranes and the respiratory system and consumption is not advised.

Slaked Lime used for chewing SBQ in PNG is thought to be causally related to squamous cell cancer of the corner of the mouth and cheek.^44^ Another characteristic of prolonged chewing of SBQ containing LAN + slaked lime is destruction of teeth. Slaked Lime contains significant amounts of Ca(OH)_2_, NaOH and trace amounts of KOH.^5^ Swedish dental studies demonstrate that exposure to KOH and strongly alkaline cleaning agents removes organic carbon from surface enamel increasing the risk of caries and dental erosion.^58^ Theoretically, replacing strongly basic slaked lime in SBQ with less basic Mg(OH)_2_ will decrease the exposure of teeth to highly basic solutions and has the theoretical potential to decrease SBQ induced teeth damage.

Magnesium is an antagonist of the physiological effects of calcium in many biological systems.^59^ If the initial stages of SBQ induced oral carcinogenesis progress through activation of cellular pathways of inflammation, as has been proposed,^6^, ^54^ then Mg^2+^ from Mg(OH)_2_ has the potential to antagonize the physiological actions of endogenous calcium while simultaneously decreasing the availability of exogenous calcium via its substitution for slaked lime in SBQ. Collectively the more benign physicochemical characteristics of Mg(OH)_2_ relative to slaked lime appear to make a clear case for harm reduction and substitution for slaked lime warrants further evaluation.

Additional chemical manipulation of the SBQ containing Mg(OH)_2_, or other test bases, could further improve its potential capacity for harm reduction. MgSO_4_ is FDA approved for human consumption.^60^ Pretreatment with MgSO_4_ has proved protective against the induction of markers of inflammation in rodent and cellular models of sepsis, lipopolysaccharide challenge and a variety of ischemic reperfusion injury models.^61^, ^62^, ^63^, ^64^ Theoretically, a very promising replacement for slaked lime in SBQ would be a mixture of predominantly Mg(OH)_2_ to facilitate absorption of physiologically active compounds and a small amount of highly soluble MgSO_4,_ to facilitate antagonism of any inflammatory actions^6^, ^54^ that require calcium.

This proof of concept pilot study focused on the least complicated form of SBQ, LAN mixed with slaked lime, as is chewed in Taiwan, Southern China, Indonesian Papua and Papua New Guinea. Experiments to determine the physiological effectiveness of replacement of slaked lime with Mg(OH)_2_, or other bases, for use with dried packet forms of SBQ and SBQ + T would be extremely interesting.

The limitations of this study are that the chemical analysis and human measurements were not a double blind study. The human measurement protocol was not designed to capture the initial hypotension to SBQ but was designed to characterize a broader temporal profile of response. In some instances, the onset of hypotension fell before or midway between measurement times and this response was missed or under reported. The responses of individuals to the two SBQ were generally similar with LAN + slaked lime invariably, but not always, greater than LAN + Mg(OH)_2._ Some of the variability in the magnitudes of the physiological responses may result from variation of levels of phyto‐compounds in the different LAN chewed during the experiments.

## 6 CONCLUSIONS

Across the Asia/Pacific region, SBQ dependence is a disease of people of low socio‐economic status^19^, ^27^, ^65^ who often live remote from hospitals and universities and, like many the world over, are frequently untrusting or fearful of interactions with these institutions. Practical quitting strategies need to be simple in design, utilize existing technologies and be sympathetic to cultural knowledge. SBQ research and treatment should not assume that strict adherence to abstinence is the only way to treat SBQ dependence. The use of non‐SBQ, such as LAN, as culturally relevant chewable placebos to satisfy abstinence‐induced cravings during attempts to abstain from chewing SBQs is a novel approach that warrants evaluation. Additionally, the effectiveness of replacing slaked lime in SBQ with orally less corrosive and chemically less reactive bases also warrants further evaluation. SBQ dependence is a disease with significant personal and social implications and treatment via abstinence offers little in the way of hope or help. This research shows that new strategies for treatment of SBQ SUD may be possible and dialogue about these strategies should be initiated.

## CONFLICT OF INTEREST STATEMENT

The authors have no financial or nonfinancial competing interests. Funding bodies play no role in the conceptualization, design, data collection, analysis, decision to publish or preparation of this manuscript.

## PATIENT CONSENT STATEMENT

All participants in this study were informed of the rational of the experiment and gave written consent to participate in the study.

## INSTITUTIONAL REVIEW BOARD STATEMENT

This study follows the principles of the Declaration of Helsinki and was performed under the ethics permit KMUH‐IRB‐20110270 issued by Kaohsiung Medical University.

## Supporting information


**Fig. S1.** UHPLC–MS/MS chromatograph of alkaloids in LAN.
A representative chromatographic separation of alkaloid and N‐nitrosoguvacoline standards and detection by UHPLC–MS/MS.
**Table S1.** Supporting Information.

**Data S1.** Supporting Information.

## Data Availability

The data that supports the findings of this study are available in the supplementary material of this article.
