# Supplementary material for: Towards a replacement therapy for stimulant betel quid dependence: A proof of concept study
Source: Addict Biol. 2024 Feb 6;29(2):e13371. doi: 10.1111/adb.13371 (PMC10898838; doi:10.1111/adb.13371)
Supplement: Supplementary file 1 — Fig. S1. UHPLC–MS/MS chromatograph of alkaloids in LAN. A representative chromatographic separation of alkaloid and N‐nitrosoguvacoline standards and detection by UHPLC–MS/MS. Table S1. Supporting Information. [file ADB-29-e13371-s002.docx]

**Reagents, Materials and standard solutions.**

Arecoline, arecaidine, guvacoline, guvacine, N-Nitrosoguvacoline and arecoline-d5 were purchased from TRC (Canada). Spectroscopy grade acetonitrile, methanol, and propanol were purchased from Avantor Inc. (USA). Formic acid (FA) was purchased from Fischer-Scientific limited (England). Ammonium sulfate and sodium sulfate was purchased from Xilong Ltd. (China). Mg(OH)_2_, Na_2_HPO_4_, K_2_HPO_4_ and C_6_H_9_Na_3_O_9_ (sodium citrate) were purchased from Shimakyu Pure Chemicals (Japan). Pure water used in the study was produced using UNISS pure water system (Taiwan).

The standard stock solutions of AN alkaloids were prepared in the methanol at 20 mg/mL concentration. Standard solutions of AN alkaloids and nitrosoguvacoline at the concentration of 1 - 1000 µg/mL were prepared daily in methanol by diluting the stock solutions of AN alkaloids. The Arecoline-d5 (internal standard, IS) solution was prepared in methanol at the concentration of 10 µg/mL. All standard curves were linear over this concentration range. All standard solutions were stored in glass bottles at 4 °C.

**UHPLC-MS/MS Instrumental Conditions**

Chromatographic analysis of AN alkaloids was carried out with a UHPLC with autosampler Shimadzu Nexera-I 2040C 3D system. Separation of AN alkaloids was achieved utilizing a C18-PFP column (150 × 4.6 mm, 3µm i.d. ACE Technologies, Scotland) and gradient elution of mobile phases (A) 0.05% FA in pure water and mobile phase (B) methanol. Column flow-rate was set at 400 µL min^-1^. The total separation run-time is 20 min. The mass analysis of AN alkaloids was carried out utilizing an LCMS-8045 mass spectrometer equipped with an electrospray-ionization source. The positive ion with multiple reaction monitoring mode was used. Nitrogen was used as a drying and heating gas at 10L/min and as the nebulizing gas at 3L/min. The heat-block temperature was 400 °C. The desolvation line temperature was 250 °C and interface temperature was 300 °C.

**S1 Table.**

UHPLC-MS/MS Instrument parameters of target alkaloids in LAN or AN extracts.

| **Analytes**  **Name** | **Molecular Weight** | **Precursor Ion (m/z)** | **Product Ion**  **(m/z)** | **Collision**  **Energy (ev)** |
| --- | --- | --- | --- | --- |
| Arecaidine | 141.17 | 142.2 | 44.10^Q^ | -18 |
|  |  |  | 42.30 | -45 |
| Guvacine | 127.14 | 128.2 | 99.10 ^Q^ | -15 |
|  |  |  | 30.20 | -17 |
| Guvacoline | 141.17 | 142.2 | 113.30 ^Q^ | -16 |
|  |  |  | 30.20 | -14 |
| Arecoline | 155.19 | 156.2 | 44.20 ^Q^ | -17 |
|  |  |  | 113.30 | -17 |
| N-nitroso guvacoline | 170.17 | 171.1 | 139.30 ^Q^ | -13 |
|  |  |  | 81.30 | -19 |
| Arecoline-d5 | 241.14 | 161.0 | 48.30 ^Q^ | -18 |
|  |  |  | 114.30 | -13 |

Note: ‘Q’ Quantification ion



**S1 Fig. UHPLC-MS/MS chromatograph of alkaloids in LAN**

A representative chromatographic separation of alkaloid and N-nitrosoguvacoline standards and detection by UHPLC-MS/MS.
