# Supplementary material for: Towards a replacement therapy for stimulant betel quid dependence: A proof of concept study
Source: Addict Biol. 2024 Feb 6;29(2):e13371. doi: 10.1111/adb.13371 (PMC10898838; doi:10.1111/adb.13371)
Supplement: Supplementary file 2 — Data S1. Supporting Information. [file ADB-29-e13371-s001.pdf]

5. Summary of Data Reported and Evaluation

5.1 Exposure data

NO evidence presented for consumption of Areca nut WITHOUT slaked lime.

Areca nut is one of the most widely used psychoactive substances with several hundred million users worldwide, predominantly in southern Asia. Areca nut can be chewed alone or in a variety of ways that differ by region.

The habit of chewing betel quid, containing fresh, dried or cured areca nut, catechu, slaked lime and flavouring ingredients wrapped in betel leaf, is widespread in India, Pakistan, Bangladesh and Sri Lanka and in migrant populations coming from these regions. Tobacco is often added. Many people in these regions chew areca nut, with a somewhat higher usage among women. Since the 1980s, the use of industrially manufactured products, often containing tobacco, has increased, especially among children and adolescents.

In Taiwan, China, the unripe areca nut is chewed with slaked lime and betel inflorescence, sometimes wrapped in betel leaf. Tobacco is not added. About 10% of the population, including adolescents, chew areca nut, with highest usage among men (90% of users) and in certain rural ethnic groups.

In parts of southern China (Hainan Island, Xiangtan), areca nut is chewed after treatment with maltose and slaked lime and is wrapped in betel leaf. Tobacco is never added. About one third of the population, both men and women, use areca nut.

In other South-East Asian countries such as Myanmar, Thailand, The Lao People's Republic, Cambodia and the Philippines, the betel quid contains areca nut, slaked lime, catechu and betel leaf. Tobacco is often added. In some of these countries, especially Thailand and Cambodia, the habit is only common among elderly women and usage is declining.

In Papua New Guinea, unripe or uncured ripe areca nut is chewed, sometimes with betel leaf, betel inflorescence or wild ginger. A stick is dipped in slaked lime and applied

to the mouth. Tobacco is never added. Over 80% of coastal inhabitants and more than 20% of highland inhabitants chew areca nut. Male and female usage is similar.

In the South Pacific island of Palau, the unripe areca nut is chewed, mixed with slaked lime, betel leaf and, frequently, tobacco. Some 80% of the population chews areca nut, and male and female usage is similar.

Betel-quid or areca-nut chewing results in exposure to areca nut alkaloids, *N*-nitroso-compounds formed from these compounds during chewing, polyphenols, trace elements and, in some cases, to tobacco.

5.2 Human carcinogenicity data

In the previous *IARC Monographs* that considered betel-quid and areca-nut chewing (Volume 37 and Supplement 7), the evidence for the carcinogenicity to humans of betel quid with tobacco was evaluated as sufficient; the evidence for betel quid without tobacco was evaluated as inadequate. Many more studies now provide evidence for the carcinogenicity of betel quid without tobacco for oral cancer and for betel quid with tobacco for cancers of the oral cavity, pharynx and oesophagus.

Oral cancer

Several case–control studies and two cohort studies reported increased risks for oral cancer for betel-quid chewing with tobacco. The statistical significance of the risk persisted after stratification for smoking and alcohol use; a strong dose–response relationship for frequency and duration of chewing was observed.

The risk for chewers of betel quid without tobacco was statistically significant in one study each from India, Pakistan and Taiwan, China, after stratifying for betel-quid chewing with tobacco, tobacco smoking and alcohol use. Several additional studies showed significant risks after stratifying or adjusting for potential confounding variables, including smoking, alcohol and human papillomavirus. A dose–response relationship for betel-quid chewing without tobacco was available in one study and was statistically significant. Suppportive evidence comes from studies that reported significant dose–response relationships for the combined categories of betel-quid chewing with and without tobacco.

Pharynx

Four studies reported results on the association of chewing betel quid with tobacco and cancer of the pharynx. In three studies that provided results stratified for smoking, the risks were significant for chewing betel quid with tobacco. One of the studies also stratified for alcohol; two studies provided significant dose–response relationships for the frequency and duration of chewing betel quid with tobacco.

Oesophagus

The risk for cancer of the oesophagus was significantly increased among chewers of betel quid in five case–control studies, four from India and one from Taiwan, China. This evidence comes from studies investigating populations that chew betel quid with and without tobacco. Significantly increased risks persisted in two studies that provided results stratified for smoking and alcohol intake. One additional study stratified for smoking only. The dose–response relationship was significant in three studies, two from India and one from Taiwan, China. The study from Taiwan, China, adjusted for smoking and alcohol.

Other cancers

Two case–control studies and one cohort study reported an association between chewing betel quid (with and without tobacco) and hepatocellular carcinoma, either stratifying or adjusting for hepatitis B and/or hepatitis C virus positivity. Similar results were seen in one case–control study for cholangiocarcinoma.

A few case–control studies were reported for cancers of the larynx, stomach, lung and cervix. The results for cancers of the stomach and cervix were suggestive of an association with chewing betel quid.

Precancerous lesions and conditions

In case–control, cross-sectional and cohort studies, chewing betel quid was strongly associated with leukoplakia. One intervention study showed decreased risk for leukoplakia in the intervention cohort. In several studies, oral submucous fibrosis was reported to occur among chewers of areca nut only, chewers of betel quid without tobacco and chewers of betel quid with tobacco, and the observed relative risk was usually extremely high. Follow-up studies showed high risks for malignant transformation of leukoplakia and oral submucous fibrosis.

5.3 Animal carcinogenicity data

Betel quid without tobacco

Betel-quid extract given by gavage produced lung adenocarcinomas in male mice. Subcutaneous injection produced fibrosarcomas at the injection site in male mice.

Cheek-pouch application of betel quid did not produce tumours in three studies in hamsters. In a fourth study, it produced carcinomas locally and in the stomach. Local application on the cheek pouch did not produce tumours in male or female baboons.

The tumour-promoting effect of betel quid was demonstrated in two studies in hamsters.

Betel quid with tobacco

Extract of betel quid combined with tobacco extract and given by gavage produced lung adenocarcinomas in male mice. Skin application produced a local squamous-cell papilloma and squamous-cell carcinomas in mice. Subcutaneous injection produced unspecified local tumours in male mice. Cheek-pouch application produced local and stomach carcinomas in hamsters, but no tumours in male or female baboons.

Areca nut

A variety of very unphysiological applications show inconsistent effects. Probably many more negative studies that did not get published.

Areca-nut extracts given by gavage produced carcinomas of the lung, liver and stomach, benign liver tumours and salivary gland tumours of an unknown nature in male mice. In another study, lung adenocarcinomas were produced.

Areca nut given in the diet produced squamous-cell carcinomas of the stomach and unspecified malignant uterine tumours in one study and oesophageal papillomas and a carcinoma in another study in male and female mice. In male hamsters, malignant lymphomas were observed. In male and female rats, no tumours were observed.

In male and female mice, oral application of areca nut produced papillomas and carcinomas in the oesophagus. In another study, skin application did not produce tumours.

Subcutaneous injection of areca-nut extract produced fibrosarcomas at the injection site in male and female mice. In another study in male mice with three types of areca-nut extract, two of the extracts produced local fibrosarcomas, liver haemangiomas, hepatomas and lung carcinomas. In a third study, local fibrosarcomas were produced in male and female rats.

Intraperitoneal injection of areca-nut extract did not produce tumours in male mice.

Cheek-pouch application of areca-nut extract produced local squamous-cell carcinomas in hamsters. In another study in females, it produced a local papilloma and a local squamous-cell carcinoma and, in a third study, local and stomach carcinomas were produced. In three other studies, no tumours were seen. hamsters

The tumour-promoting effect of areca nut was demonstrated in one study in mice, one study in rats and three studies in hamsters. Five positives and many negatives ?

Areca nut with tobacco

Skin application of areca-nut extract with tobacco extract produced local papillomas and squamous-cell carcinomas in male and female mice.

In three studies, cheek-pouch application of areca-nut and tobacco extract in hamsters produced local squamous-cell carcinomas in males, stomach tumours in males, and local and stomach carcinomas, respectively. No tumours were seen in two other studies.

Areca nut with slaked lime

In one study, areca nut with slaked lime in the diet produced papillomas in the oral cavity and the forestomach, and carcinomas in various major organs in male and female rats. In another study, no tumours were produced.

Areca nut with tobacco and slaked lime

Cheek-pouch application of areca nut with slaked lime and tobacco produced local and stomach carcinomas in hamsters.

Areca nut with betel leaf

Areca-nut extract with betel-leaf extract given by gavage produced lung adenocarcinomas in male mice. It did not produce tumours when given subcutaneously to male mice or applied to the cheek pouch of male and female hamsters.

Betel leaf

Beneficial antioxidant effect or since leaf pH is 4 possibly, extra buffering strength at low acidic pH?

Betel-leaf extract given by gavage in two studies or intraperitoneally to male mice, in the diet to male and female rats or by cheek-pouch application to hamsters in two studies did not produce tumours.

Betel leaf also showed some antitumorigenic activity in three studies in mice, in one study in rats and in three studies in hamsters.

Pan masala

Pan masala in the diet produced benign and malignant tumours in various organs, most frequently adenocarcinoma of the lung, in male and female mice. *Pan masala* extract did not produce tumours when administered by gavage to male and female mice, or when applied orally to rats in another study.

The tumour-promoting effect of *pan masala* was demonstrated in one study in mice. The number of studies investigating this substance in animals is limited.

Arecoline

Arecoline given by gavage produced lung adenocarcinomas, stomach squamous-cell carcinomas and liver haemangiomas in male mice. It did not produce tumours when given by gavage to female mice or in the drinking-water to male and female hamsters, when injected subcutaneously into male mice or when administered intraperitoneally to male mice.

No tumours were produced by local intraoral painting in rats.

No initiating effect of arecoline was demonstrated in one study in male hamsters.

Arecoline with slaked lime

Cheek-pouch application of arecoline following application of slaked lime produced an oesophageal papilloma in female hamsters. In two studies in male and female hamsters, no tumours were seen after administration in the diet or in drinking-water.

Slaked lime

Slaked lime did not produce tumours in male or female mice when given by gavage, in male or female hamsters when given in the drinking-water or diet or in three cheek-pouch application studies in hamsters.

No promoting effect for slaked lime was shown in male and female hamsters.

Arecaidine alone

Local application of arecaidine to the cheek pouch did not produce tumours in male hamsters.

The tumour-promotion effect of arecaidine was demonstrated in one study in hamsters.

5.4 Other relevant data

Areca nut should be replaced with SBQ otherwise this statement is factually completely incorrect.

Areca nut is chewed for its psychostimulating effects, such as relaxation, improved concentration and enhanced satisfaction after eating. A majority of users reported the development of tolerance to these effects. Withdrawal symptoms on trying to quit the habit were mood swings, anxiety, irritability, reduced concentration, sleep disturbance and craving. These findings are regarded to be consistent with the existence of a dependence syndrome among regular users. In rare cases, areca nut psychosis has been reported to occur in heavy users following abrupt cessation of the habit.

Human studies

Powdered areca nut placed in the oral cavity of human volunteers gives rise to the rapid appearance of arecoline in blood plasma, indicating systemic absorption of this alkaloid. Peak concentrations of arecoline in blood plasma have been measured in human subjects 5–10 h after application of a dermal dose.

Eugenol and dihydroxychavicol, metabolites of safrole, are found in the urine of betel-quid chewers.

The areca-nut-derived nitrosamines, *N*-nitrosoguvacoline and 3-(methylnitroso)-propionitrile, have been detected in saliva and are most probably produced *in situ* during betel-quid chewing. This nitrosation reaction can be mimicked *in vitro*. In addition, the volatile nitrosamines, *N*-nitrosodimethylamine and *N*-nitrosodiethylamine, and the tobacco-specific nitrosamines, *N*-nitrosonornicotine, 4-(methylnitrosamino)-1-(3-pyridyl)-1-butanone and N'-nitrosoanabasine, are present in the saliva of chewers of betel quid with tobacco. Endogenous nitrosation has been demonstrated in chewers of betel quid mixed with proline, by measuring *N*-nitrosoproline in saliva and urine.

Formation of reactive oxygen species in the oral cavity during betel-quid chewing has been demonstrated. In-vitro studies have shown that the generation of reactive oxygen species is due to auto-oxidation of the polyphenols in areca nut and catechu. This reaction is enhanced by alkaline pH (from the slaked lime) and by the presence of the transition metals, copper and iron.

Areca-nut chewing has significant effects on the hard and soft tissues of the oral cavity. Although some studies have described a protective effect of areca-nut chewing on dental caries, the habit causes severe tooth wear and may also enhance gingivitis. Areca-nut polyphenols increase cross-linkage of collagen and inhibit the activity of collagenase.

Arecoline modulates the activity of matrix metalloproteinases, their tissue inhibitors and lysyl oxidase, which leads to the accumulation of collagen in the fibroblasts of the oral mucosa. This may result in the development of submucous fibrosis. The significant amounts of soluble copper released into the oral cavity by areca-nut chewing may further contribute to the development of this condition.

Betel-quid chewing by pregnant women has been associated with adverse pregnancy outcomes including reduction in birth weight, pre-term delivery, stillbirth and fetal malformation.

Confusing areca nut with SBQ.

The alkaloids in areca nut have antimuscarinic effects. Areca-nut chewing increases the blood plasma concentrations of adrenaline and noradrenaline. Arecoline and betel-quid use increase occipital α activity and generalized β activity in electroencephalograms. Betel-nut chewing has antidepressant effects and increases heart rate and blood flow through carotid arteries.

Areca-nut chewing is said to soothe digestion and enhances the production and secretion of saliva.

Betel-quid chewing aggravates asthma by reducing the forced expiratory volume. Arecoline can cause constriction of bronchial smooth muscle tissue *in vitro* and bronchoconstriction in betel-quid users with asthma.

Although short-term hypoglycaemic effects have been reported, areca nut can clearly cause chronic hyperglycaemia. The alkaloids in areca nut block the receptor of γ-aminobutyric acid, which increases the release of glucagon. Areca-nut use has been shown to be an independent risk factor for type-2 diabetes.

Areca-nut chewing increases skin temperature, pulse rate and — in novice users — blood pressure.

Chewing SBQ will induce this physiology but areca nut does not, confusion yet again

Elevated micronucleus formation and chromosome breaks have been reported in oral exfoliated cells in chewers of betel quid with or without tobacco. Micronucleus formation has been observed in precancerous lesions in the oral cavity of chewers.

Elevated sister chromatid exchange and micronucleus formation have been demonstrated in cultured peripheral lymphocytes collected from chewers of *pan masala* and areca nut with and without tobacco and slaked lime.

TP53 mutations were infrequent or absent in oral premalignant lesions and squamous-cell carcinomas in subjects chewing betel quid without tobacco, but accumulation of p53 protein was observed. *TP53* or *ras* mutations, p53 or ras protein accumulation and a number of other gene/protein alterations were observed in premalignant lesions and squamous-cell carcinomas of chewers of betel quid with tobacco.

No clear gene–environment interaction could be established for polymorphisms in carcinogen metabolic enzymes because of the presence of concurrent confounding habits of tobacco chewing or smoking or alcohol consumption in cases.

Elevated COX2 protein levels have been detected in ‘moderate human submucous fibrosis’ tissue by immunohistochemistry.

Animal studies

In rats, the major metabolic pathway of arecoline is via de-esterification and production via conjugated mercapturic acid. In-vitro data suggest that arecoline is metabolized by carboxylesterase (EC 3.1.1.1) in mouse liver and kidney.

Male Swiss albino mice fed areca-nut powder or arecoline showed enhanced levels of the hepatic cytochromes P450 and b_5 and decreased levels of hepatic glutathione. When given orally to these mice, betel-leaf extract enhanced hepatic superoxide dismutase activity, increased vitamin A and C concentrations in the liver and inhibited catalase activity.

Pan masala reduced testis weight in mice and enhanced the frequency of morphological abnormalities in mouse sperm. Areca-nut extracts were embryotoxic when given to pregnant mice during early gestation, and teratogenic when administered into the yolk sac of chick embryos. Extracts of *Piper betle* stalks disturbed the estrus cycle in female rats and reduced fertility in males. In mice, arecoline was genotoxic to early spermatids and it increased the frequency of abnormal sperm.

The diabetogenic effects of areca nut were produced in mice fed ground areca nut and were observed in subsequent generations not fed betel quid. Not a strong effect, see above.

Arecoline caused inhibition of both humoral and cell-mediated immune responses in mice.

At higher doses, betel-quid extracts reduce the activity of hepatic glutathione peroxidase and glutathione *S*-transferase.

In rats, *pan masala* impaired liver function and decreased relative weights of gonads and brain. Areca nut and areca-nut ingredients influenced the rate of RNA and DNA synthesis in various tissues of treated mice. Effects on gastrointestinal tract function and control of the cardiovascular system have been described for areca nut and its ingredients in a number of experimental models, both *in vivo* and *in vitro*. In general, the effects observed correspond to those seen in regular users of areca nut and betel quid.

In-vitro studies

results will be strongly influenced by pH of culture medium given pKa of arecoline

Glutathione depletion and reduction of glutathione *S*-transferase activity have been demonstrated in cultured human oral keratinocytes and in fibroblasts treated with arecoline.

Areca-nut extract enhanced COX2 expression and prostaglandin E_2 production in cultured human gingival keratinocytes and human buccal mucosa fibroblasts.

Phytohaemagglutinin-stimulated proliferation of human lymphocytes *in vitro* was inhibited by areca-nut extract.

Extracts (water, water/ethanol, dimethyl sulfoxide) of *pan masala* were weakly mutagenic in bacteria, but induced chromosomal aberrations, sister chromatid exchange and micronucleus formation in Chinese hamster ovary cells in the absence of metabolic activation. Sister chromatid exchange and sperm abnormalities were induced by these extracts in mice *in vivo*.

Extracts of betel quid with or without tobacco were mutagenic in bacteria, but only extracts with tobacco induced mutations in Chinese hamster V79 cells. Betel-quid extracts without tobacco were weak inducers of sister chromatid exchange in Chinese

hamster ovary-K1 cells *in vitro* and did not give rise to micronucleus formation in mouse bone marrow.

Aqueous extracts of areca nut produced gene conversion in yeast, DNA strand breaks in mouse kidney cells, and gene mutation, chromosomal aberrations, sister chromatid exchange and micronucleus formation in Chinese hamster ovary cells *in vitro*. These extracts also induced chromosomal aberrations and cell transformation in mouse C3H 10T1/12 cells. Areca-nut extracts induced DNA strand breaks and DNA–protein cross-links in cultured human primary buccal epithelial cells, DNA strand breaks in human oral mucosal fibroblasts and unscheduled DNA synthesis in Hep2 human laryngeal carcinoma cells and in primary human gingival keratinocytes. Sister chromatid exchange and micronucleus formation were seen in bone-marrow cells of mice treated with these extracts *in vivo*. Micronuclei were also induced in cheek-pouch epithelial cells of hamsters treated *in vivo*, with or without slaked lime, and in fibroblasts of healthy subjects and ataxia telangiectasia patients. All these effects of areca-nut extracts were seen without metabolic activation.

Arecoline and other areca-nut alkaloids gave positive responses in most bacterial mutagenicity assays, and induced chromosomal aberrations, micronucleus formation and sister chromatid exchange in mammalian cells, both *in vitro* and *in vivo*.

Data on the genotoxicity of aqueous extracts of *Piper betle* L. inflorescence and of betel leaf are limited, and generally negative. Inflorescence was reported to induce DNA strand breaks in cultured human oral mucosa fibroblasts in one study.

Safrole and eugenol did not induce mutation in bacteria, whereas hydroxychavicol gave inconsistent results. However, the latter compound did induce chromosomal aberrations and micronucleus formation in Chinese hamster ovary cells.

Catechu was not mutagenic in bacteria.

5.5 Evaluation

There is *sufficient evidence* in humans for the carcinogenicity of betel quid with tobacco. Betel quid with tobacco causes oral cancer and cancer of the pharynx and oesophagus.

There is *sufficient evidence* in humans for the carcinogenicity of betel quid without tobacco. Betel quid without tobacco causes oral cancer.

There is *sufficient evidence* in experimental animals for the carcinogenicity of betel quid without tobacco.

There is *sufficient evidence* in experimental animals for the carcinogenicity of betel quid with tobacco. ***No Mention of carcinogenicity in humans for Areca nut.

There is *sufficient evidence* in experimental animals for the carcinogenicity of areca nut.

There is *sufficient evidence* in experimental animals for the carcinogenicity of areca nut with tobacco.

There is *limited evidence* in experimental animals for the carcinogenicity of arecoline.

There is *inadequate evidence* in experimental animals for the carcinogenicity of arecaidine.

There is *evidence suggesting lack of carcinogenicicy* in experimental animals for betel leaf.

There is *evidence suggesting lack of carcinogenicicy* in experimental animals for slaked lime.

Overall evaluation

Betel quid with tobacco is *carcinogenic to humans (Group 1)*.

Betel quid without tobacco is *carcinogenic to humans (Group 1)*.

Areca nut is *carcinogenic to humans (Group 1)*.

In reaching the latter conclusion, the Working Group noted that a common component of all betel-quid preparations is the areca nut. This evaluation is based on strong evidence that areca nut causes oral submucous fibrosis, a precancerous condition in humans, and sufficient evidence of carcinogenicity in experimental animals. In addition, there is strong supporting evidence for this conclusion.
